# Prognostic Role of Uric Acid-Related Gene Signatures in Glioblastoma Multiforme: Insights from Bulk RNA and Single-Cell RNA Sequencing

**DOI:** 10.3390/cancers18081297

**Published:** 2026-04-20

**Authors:** Kai Sun, Chao Li, Jiangting Wang, Ruxiang Xu

**Affiliations:** 1 Department of Neurosurgery, Sichuan Provincial People’s Hospital, University of Electronic Science and Technology of China, Chengdu 611731, China; sunkai@med.uestc.edu.cn; 2 Department of Hepatobiliary Surgery, Zhuji Affiliated Hospital of Wenzhou Medical University, Zhuji 311800, China; lichao123@stu.kust.edu.cn; 3 Department of Anesthesiology, Xiangyang Central Hospital, Affiliated Hospital of Hubei University of Arts and Science, Xiangyang 441000, China

**Keywords:** glioblastoma multiforme, uric acid, bulk RNA sequencing, single-cell RNA sequencing, prognostic signature

## Abstract

Background: Among primary intracranial neoplasms in adults, glioblastoma multiforme stands out for both its prevalence and its exceptionally invasive character. Uric acid-related genes (UARGs) may enhance tumor cell invasiveness and drug resistance by promoting oxidative stress responses. This study aimed to elucidate uric acid-driven mechanisms in glioblastoma, focusing on risk stratification and therapeutic vulnerability. Methods: Transcriptomic profiles of GBM were retrieved from TCGA and GEO repositories, followed by performing differentially expressed analysis, univariate Cox and LASSO regression, in order to screen prognostic UARGs and construct a risk model. Then, prognostic analyses were expanded by performing immune microenvironment analysis, drug sensitivity analysis, tumor mutation analysis, independent prognostic analysis, and nomogram construction. Additionally, dataset GSE162631 was interrogated to pinpoint pivotal cell subsets and to map intercellular communication as well as pseudo-time analysis. Results: A risk model incorporating six prognostic UARGs (TIMP1, PLAUR, CTSB, KLF10, RARRES2, and PTPRN) was constructed and identified as a favorable prognostic signature. Resting dendritic cells and drugs (including acetalax and trametinib) were found to be associated with GBM patients’ risk stratification. Low-risk patients showed relatively higher mutation rates of PTEN and TP53. A nomogram was developed based on RARRES2 and PTPRN, which exhibited favorable predictive performance for GBM prognosis. Furthermore, scRNA-seq profiling identified dendritic cells (DCs), macrophages, and T cells as key populations in the tumor microenvironment. Intercellular communication inference indicated relatively strong DCs-macrophage crosstalk, and pseudo-time analysis linked prognostic UARG expression to the differentiation trajectory of critical cell subsets. Conclusions: This study identified uric acid-related genes as potential independent indicators of clinical outcomes in glioblastoma progression. A novel prognostic UARG-associated signature was developed and validated, which showed potential in predicting GBM patient outcomes.

## Simple Summary

Glioblastoma multiforme (GBM) stands out among primary adult intracranial neoplasms for its prevalence and exceptionally invasive character. Uric acid-related genes (UARGs) may enhance tumor cell invasiveness and drug resistance by promoting oxidative stress responses. This study aimed to elucidate uric-acid-driven mechanisms in GBM, focusing on risk stratification and therapeutic vulnerability. Through integrative analysis of bulk RNA and single-cell RNA sequencing data from TCGA, GEO, and GSE162631 repositories, we constructed and validated a novel prognostic signature incorporating six pivotal UARGs (TIMP1, PLAUR, CTSB, KLF10, RARRES2, and PTPRN). Our findings linked this signature to resting dendritic cells, drug sensitivities including acetalax and trametinib, and mutation profiles of PTEN and TP53. Intercellular communication analysis indicated strong dendritic cell-macrophage crosstalk, with prognostic UARG expression mapped to differentiation trajectories of critical cell subsets, offering valuable insights for predicting outcomes and guiding GBM research and treatment.

## 1. Introduction

Glioblastoma multiforme (GBM), designated WHO Grade IV, represents the most frequent and most malignant primary intracranial neoplasm in adults [1]. Despite standardized treatment strategies, the mortality rate remains exceedingly high, and fewer than 5% survive beyond five years [2]. Furthermore, recurrence is nearly inevitable, with approximately 70% of patients experiencing tumor relapse within 6 to 8 months post-surgery, highlighting the significant challenge posed by its high recurrence rate and the urgent need for more effective therapeutic approaches [3]. GBM is treated through a multimodal approach involving maximal surgical resection, concurrent chemoradiotherapy with temozolomide, and adjuvant therapies, yet its highly invasive nature and rapid development of resistance severely limit treatment efficacy [4]. The tumor microenvironment is immunosuppressive, with dense extracellular matrix and a blood–brain barrier that restricts immune cell infiltration and therapeutic delivery. Additionally, intratumoral heterogeneity, characterized by diverse genetic mutations and phenotypes, leads to variable treatment responses and the emergence of resistant clones, further complicating therapeutic strategies [5]. Therefore, the systematic characterization of newly uncovered therapeutic targets and prognostic biomarkers is imperative to improve the prognosis of GBM patients [6]. This mandates a comprehensive elucidation of the molecular pathogenesis underpinning GBM and holds the potential to provide patients with more effective therapeutic options and enhanced survival outcomes.

Among various metabolic alterations in GBM, emerging evidence suggests that uric acid metabolism may function as a key driver of tumor progression, yet its precise mechanisms remain largely unexplored. Uric acid (UA) represents the final catabolite of purine metabolism, primarily generated through the oxidation of purine bases (such as adenine and guanine) by the enzyme xanthine oxidase [7,8]. It serves as a potent antioxidant, scavenging free radicals and chelating transition metal ions, thereby protecting cells from oxidative damage and contributing to the maintenance of redox homeostasis [9]. However, the role of UA in cancer appears paradoxical, as accumulating evidence suggests that UA and uric acid-related genes (UARGs) have been implicated in the pathogenesis and progression of GBM [10]. While UA may exert protective effects under physiological conditions, in the tumor microenvironment, UA crystals can activate inflammasomes, promoting chronic inflammation and tumor microenvironment remodeling, which may facilitate tumor growth and invasion [11]. Additionally, UARGs may modulate intracellular redox balance, instigating the production of reactive oxygen species (ROS) via xanthine oxidase, which can influence cell proliferation and apoptosis [12]. These mechanisms highlight the potential role of UA in GBM biology and suggest that targeting UA pathways could offer therapeutic benefits. However, the precise mechanisms by which UARGs contribute to GBM progression and drug resistance remain incompletely understood, constituting a pivotal void in current understanding that limits the development of UARG-targeted therapeutic strategies.

Given the profound cellular heterogeneity of GBM and the multicellular and highly dynamic network among diverse constituents within the tumor microenvironment, comprehensive characterization of UARG expression patterns at single-cell resolution is essential. Single-cell RNA sequencing (scRNA-seq) is a transformative technology that enables the dissection of genetic sequences at single-cell resolution, thereby delineating heterogeneous cell-state landscapes and transitions with unprecedented resolution [13]. Compared with traditional bulk sequencing, scRNA-seq can detect cellular subtypes and gene expression changes that may be obscured in bulk analysis, offering unprecedented resolution to dissect complex biological processes [14,15]. In GBM research, this technology has proven particularly valuable for identifying distinct cellular populations, including tumor cells, immune cells, and stromal cells, as well as elucidating their dynamic interactions within the tumor microenvironment and the molecular mechanisms driving disease progression [16,17,18]. This high-resolution analysis not only reveals key cell types and pathways involved in GBM pathogenesis but also highlights potential therapeutic targets and biomarkers, thereby accelerating the creation of precision therapies with enhanced efficacy. ScRNA-seq not only uncovers discrete cellular constituents embedded in the tumor microenvironment but also elucidates the intricate interactions among these cells; ScRNA-seq not only enables the identification of distinct cellular populations, including neoplastic cells, immunocytes, and vascular endothelial cells, but also elucidates the intricate interactions among these cells within the tumor microenvironment [15]. In clinical development, scRNA-seq can facilitate the identification of prognostic UARGs with enhanced precision, thereby providing a basis for patient stratification and refined surveillance of treatment efficacy and disease trajectory [19].

Despite the recognized importance of UARGs in GBM and the availability of advanced technologies like scRNA-seq, a systematic and comprehensive analysis integrating bulk and single-cell transcriptomics to characterize UARG-based prognostic signatures remains lacking. In this study, we bridge prevailing knowledge voids by constructing a novel UARG-based risk framework informed by bulk and single-cell transcriptome data from public databases such as The Cancer Genome Atlas (TCGA) (http://cancergenome.nih.gov/, accessed on 3 April 2025) and Gene Expression Omnibus (GEO) (http://www.ncbi.nlm.nih.gov/geo/, accessed on 3 April 2025). The prognostic performance of the model was independently verified in external cohorts, followed by a comprehensive suite of bioinformatics interrogations, including immunomicroenvironment characterization, drug sensitivity profiling, and tumor mutation analysis. Additionally, leveraging scRNA-seq, we charted the GBM microenvironment, quantified cell-type-specific risk signatures, and decoded intercellular communication hubs, yielding actionable therapeutic leads and refined treatment trajectories.

## 2. Materials and Methods

### 2.1. Data Collection

Transcriptome data of glioblastoma (GBM) patients were obtained from The Cancer Genome Atlas (TCGA) database (http://cancergenome.nih.gov/, accessed on 3 April 2025). TCGA-GBM, designated as Training Set 1, included tumor tissue samples from 165 GBM patients (GBM group), among which 153 samples had a survival time of over 60 days. Normal brain tissue samples (*n* = 1148) were retrieved from the Genotype-Tissue Expression (GTEx) portal (https://gtexportal.org, accessed on 3 April 2025) to serve as the normal control group, and these samples were also defined as Training Set 2. External validation transcriptome data and clinical information of GBM cases were acquired from the Chinese Glioma Genome Atlas (CGGA) portal (http://www.cgga.org.cn, accessed on 3 April 2025). This validation set contained 656 GBM tumor samples, and 650 eligible samples (with survival time > 60 days) were selected for risk model validation. Single-cell RNA sequencing (scRNA-seq) data of GBM from the GSE162631 dataset (platform: GPL24676) were downloaded from the Gene Expression Omnibus (GEO) database (https://www.ncbi.nlm.nih.gov/geo/, accessed on 3 April 2025); this dataset included 4 GBM tumor tissue samples and 4 peritumoral tissue samples as controls. Additionally, a total of 3632 uric acid-related genes (UARGs) [20] (Table S1) were obtained from the Genecards database (https://www.genecards.org/, accessed on 3 April 2025). All aforementioned data were downloaded on 3 April 2025 and formatted uniformly prior to subsequent analyses.

### 2.2. Comprehensive Identification and Functional Annotation of Differentially Expressed Uric Acid-Related Genes (DE-UARGs)

Differentially expressed genes (DEGs) were identified between GBM and normal groups in the TCGA-GBM dataset and Training Set 2 using the DESeq2 package (v 1.40.2) [21] (|log2 fold change (FC)| > 1 and adj. *p* < 0.05). Subsequently, volcano plots and heatmaps were generated using the ggplot2 (v 3.5.1) [22] and ComplexHeatmap (v 2.16.0) [23] packages, respectively. Differentially expressed uric acid-related genes (DE-UARGs) were defined as the intersection of UARGs and DEGs, and this overlap was quantified and visualized using the ggvenn package (v 1.2.2) [24]. To systematically explore the biological functions and pathways of DE-UARGs in GBM, Gene Ontology (GO) and Kyoto Encyclopedia of Genes and Genomes (KEGG) enrichment analyses were performed using the clusterProfiler package (v 4.15.0.3) (adj. *p* < 0.05) [25]. GO enrichment analysis included three categories: biological process (BP), cellular component (CC), and molecular function (MF). Furthermore, protein–protein interaction (PPI) networks of proteins encoded by DE-UARGs were constructed using the STRING database (http://www.string-db.org/, accessed on 3 April 2025) with a confidence score threshold of ≥0.9, to investigate the interaction relationships between these proteins.

### 2.3. Development and Validation of UARG-Based Prognostic Signature

Using the coxph function of the survival package (v 3.7.0) [26], the DE-UARGs in TCGA-GBM specimens were subjected to univariate Cox regression analysis (HR ≠ 1, *p* < 0.001) and proportional hazards assumption testing (*p* > 0.05) to identify candidate prognostic UARGs. To further develop the risk model while avoiding overfitting, LASSO regression (10-fold cross-validation) was implemented via the glmnet package (v 4.1.8) [27] to select prognostic UARGs for model construction.

Subsequently, TCGA-GBM tissue samples with complete survival information were independently stratified into the high-risk group (HRG, risk score ≥ optimal cutoff value, determined by the surv_cutpoint function) and low-risk group (LRG, risk score < optimal cutoff value). Kaplan–Meier survival analysis (log-rank test, *p* < 0.05) and receiver operating characteristic (ROC) curves were generated using the survival (v 3.7.0) and timeROC (v 0.4) package [28], respectively, to evaluate the prognostic performance of the risk model. The risk model was considered to have moderate discriminatory power if its area under the curve (AUC) for 1-, 2-, and 3-year overall survival all exceeded 0.6. In addition, the risk prediction model was validated in the CGGA validation set using identical grouping criteria and evaluation metrics to ensure its robustness and predictive accuracy.

To evaluate the predictive performance of the risk model constructed by the six prognostic UARGs identified in this study, five-gene risk models associated with cuproptosis that were recently published [29] were selected as comparators. Analyses were performed in two independent cohorts: TCGA-GBM and CGGA. First, risk scores for both the UARG model and the cuproptosis-related model were calculated according to the regression coefficients reported in the literature. The TCGA cohort was used as the training set, and the optimal cutoff value was determined using the surv_cutpoint function, by which patients were stratified into high- and low-risk groups. Next, survival differences between the high- and low-risk groups were compared using Kaplan–Meier curves with the log-rank test (*p* < 0.05). Time-dependent ROC curves were applied to assess the 1-, 2-, and 3-year AUC values of the two models. Hazard ratios (HRs) with 95% confidence intervals (CIs) and log-rank *p*-values were calculated for each model and visualized using forest plots and bar plots. Finally, subgroup analyses stratified by age (≤60 years vs. >60 years) and gender were conducted in the TCGA cohort to validate the generalizability of the model.

### 2.4. Profiling of the Immune Microenvironment

The progression and therapeutic resistance of GBM are intrinsically associated with substantial intratumoral immune compartment heterogeneity [4]. The infiltration abundances of 22 immune cell subsets were deconvoluted from transcriptome data using the CIBERSORT algorithm [30], and differential immune cells (DICs) were identified via the Wilcoxon test (*p* < 0.05).

ESTIMATE scores, immune scores, stromal scores, and tumor purity were calculated between the HRG and LRG using the ESTIMATE algorithm (https://bioinformatics.mdanderson.org/estimate/, accessed on 3 April 2025), with statistical differences assessed by the Wilcoxon test (*p* < 0.05).

### 2.5. Drug Sensitivity Analyses

Management recommendations for GBM were derived from a drug sensitivity analysis conducted with the Genomics of Drug Sensitivity in Cancer (GDSC) database (https://www.cancerrxgene.org/, accessed on 3 April 2025). The half-maximal inhibitory concentration (IC50) of 251 common chemotherapeutic and molecular-targeted agents in TCGA-GBM was computed with the pRRophetic package (v 0.5) [30] to infer drug sensitivity. Variations in GBM-directed medication sensitivity between the HRG and LRG were contrasted by the Wilcoxon test (*p* < 0.05). The top 10 drugs showing the most prominent disparities were selected and plotted in a box plot.

### 2.6. Tumor Mutation Analysis

Tumor mutation burden (TMB), defined as the density of non-synonymous mutations per megabase in the tumor genome, reflects tumor genetic instability [31]. Mutation annotation files of TCGA-GBM were processed using the maftools package (v 2.16.0) [32] to calculate TMB scores for the high-risk and low-risk GBM subgroups. Somatic mutation profiles of HRG and LRG in TCGA-GBM were analyzed using the same package (v 2.16.0) to explore intergroup variations. Differential mutation patterns between the two risk strata were identified using DESeq2 (v 1.40.2), and differentially mutated genes (DMGs) were screened (adj. *p* < 0.05, |log2FC| > 1). Univariate Cox regression analysis (HR ≠ 1, *p* < 0.05) and proportional hazards (PH) assumption testing (*p* > 0.05) were performed on these DMGs using the survival package (v 3.7.0) to select key mutated genes, whose mutation sites were visualized as lollipop plots. Finally, the mutation status of prognostic UARGs was investigated via mutation analysis using GBM data retrieved from the cBioPortal database (https://www.cbioportal.org/, accessed on 3 April 2025).

### 2.7. Independent Prognostic Analysis and Nomogram

Multivariate Cox regression analysis (HR ≠ 1, *p* < 0.05) and proportional hazards assumption tests (*p* > 0.05) were performed using the survival package (v 3.7.0) to evaluate whether prognostic UARGs served as independent predictors in TCGA-GBM cases with complete survival annotations. A nomogram incorporating independent prognostic variables was constructed using the rms package (v 6.8.1) [33] to estimate 1-, 2-, and 3-year overall survival probabilities for GBM patients. Total points derived from the nomogram were used to predict survival probabilities of GBM patients. Calibration curves for evaluating nomogram reliability were generated using the rms package (v 6.8.1). Decision curve analysis (DCA), a straightforward and robust method for assessing clinical prediction models, diagnostic assays, and molecular markers, was conducted using the ggDCA package (v 1.1) to generate decision curves and evaluate the clinical utility of the nomogram. ROC curves were generated using the survivalROC package (v 1.0.3.1) [34], and an area under the curve (AUC) value > 0.7 indicated favorable predictive performance of the nomogram.

### 2.8. The scRNA-seq Data Processing

In all samples of the GSE162631 dataset, scRNA-seq data were filtered using the PercentageFeatureSet function of the Seurat package (v 5.1.0) [35] to select high-quality cells, with filtering criteria set as: nFeature_RNA (200–4000), nCount_RNA (200–20,000), mitochondrial gene proportion < 15%, and genes expressed in at least 3 cells. After data normalization via the NormalizeData function, the top 2000 highly variable genes (HVGs) were extracted using the FindVariableFeatures function. Subsequently, the runPCA, Jackstraw, and ElbowPlot functions were applied to principal component analysis (PCA) outputs to identify significant top principal components (PCs) (*p* < 0.05). Batch effects were eliminated using Canonical Correlation Analysis (CCA), and Uniform Manifold Approximation and Projection (UMAP) was performed via the RunUMAP function (resolution = 0.5) to determine cell clusters. Cell types were annotated based on marker genes using the FindAllMarkers function (logfc.threshold = 0.25, min.pct = 0.1, only.pos = TRUE) [36054938], and the expression of marker genes across annotated cell types was visualized as bubble plots. Functional enrichment analysis of annotated cells was performed using irGSEA (v 2.1.5) to characterize the biological pathways driving GBM development [36].

Furthermore, a stacked bar chart illustrating the relative abundance of each annotated cell type across samples was generated using ggplot2 (v 3.5.1). The expression patterns of prognostic UARGs in these annotated cell types were then investigated. Additionally, differences in cell type distributions between GBM and normal tissues were analyzed using the Wilcoxon test (*p* < 0.05). Annotated cell types with the most significant differences in prognostic UARG expression between GBM and normal tissues were defined as key cell populations (Wilcoxon test, *p* < 0.05).

### 2.9. Cell Communication and Pseudo-Time Analysis

The CellChat package (v 1.6.0) [37] was applied to analyze cell–cell communication in the GSE162631 dataset. The intensity of signal transduction across signaling pathways was visualized as heatmaps, and a receptor–ligand interaction bubble plot was generated to display interactions between key cell populations.

In addition, key cells underwent a secondary round of HVG screening, dimensionality reduction, and clustering following the method described in Section 2.8. Subsequently, the key cells were annotated into different subgroups, and the monocle package (v 2.28.0) [38] to characterize their differentiation states and subgroup-specific trajectories. The expression dynamics of prognostic UARGs were tracked along the differentiation trajectories of key cell populations.

### 2.10. Cell Culture

Normal human astrocytes (NHAs) and several human glioblastoma cell lines were furnished by the cell bank of the Chinese Academy of Sciences in Shanghai, China. The cell lines consisted of U87MG, U251, and LN229. Cell lines of glioblastoma were grown in Dulbecco’s Modified Eagle’s Medium that had a high level of glucose (Product No. 21013024, Gibco, Waltham, MA, USA). To this culture medium, 10% Fetal Bovine Serum (Product No. A5670701, Gibco, USA) and 1% penicillin/streptomycin (Product No. 15140122, Gibco, USA) were added. Conversely, NHA cells were incubated in astrocyte medium (Product No. #1801, ScienCell, Carlsbad, CA, USA). Each individual cell was maintained at a temperature of 37 degrees Celsius within a humidified incubator that had an atmosphere consisting of 5% carbon dioxide.

### 2.11. Reverse Transcription-Quantitative PCR (RT-qPCR) Assay

According to the manufacturer’s guidelines, the TRIzol™ Reagent (15596026CN, Invitrogen, Carlsbad, CA, USA) was used to extract ribonucleic acid (RNA) from clinical tissue specimens and cells cultured in the laboratory. The All-in-One™ miRNA First-Strand cDNA Synthesis Kit 2.0 (Product Code QP113, produced by GeneCopoeia in Guangzhou, China) was utilized to transform the extracted ribonucleic acid (RNA) into complementary deoxyribonucleic acid (cDNA) via reverse transcription. Subsequently, the PowerTrack™ SYBR Green Master Mix, which is intended for quantitative polymerase chain reaction (qPCR) (Product Code A46109, provided by Thermo Fisher Scientific in the Waltham, MA, USA), was applied for quantification on the Roche LightCycler480 system (Product Code 900066, made by Roche in Basel, Switzerland). Glyceraldehyde 3-phosphate dehydrogenase (GAPDH), functioning as a constitutive gene, was employed to standardize the expression of TIMP1, PLAUR, CTSB, KLF10, RARRES2, and PTPRN. The primer sequences are listed in Table 1.

**Table 1:** Primer sequences for prognostic UARGs.

Genes	Forward Primer (5′-3′)	Reverse Primer (5′-3′)
GAPDH	GTCTCCTCTGACTTCAACAGCG	ACCACCCTGTTGCTGTAGCCAA
TIMP1	GGAGAGTGTCTGCGGATACTTC	GCAGGTAGTGATGTGCAAGAGTC
PLAUR	CCACTCAGAGAAGACCAACAGG	GTAACGGCTTCGGGAATAGGTG
CTSB	GCTTCGATGCACGGGAACAATG	CATTGGTGTGGATGCAGATCCG
KLF10	AGGAGTCACATCTGTAGCCACC	GAACGGGCAAACCTCCTTTCAC
RARRES2	GAAACCCGAGTGCAAAGTCAGG	CCGCAGAACTTGGGTCTCTATG
PTPRN	TGGAGATCCTGGCTGAGCATGT	GGTCACATCAGCCAAAGACAGG

### 2.12. Statistical Analysis

All data were processed using the R software (v 4.3.3). Comparisons were performed using the Wilcoxon test, and statistical significance was defined as *p* < 0.05.

## 3. Results

### 3.1. Identification and Functional Assessment of 880 DE-UARGs

**Figure 1:**
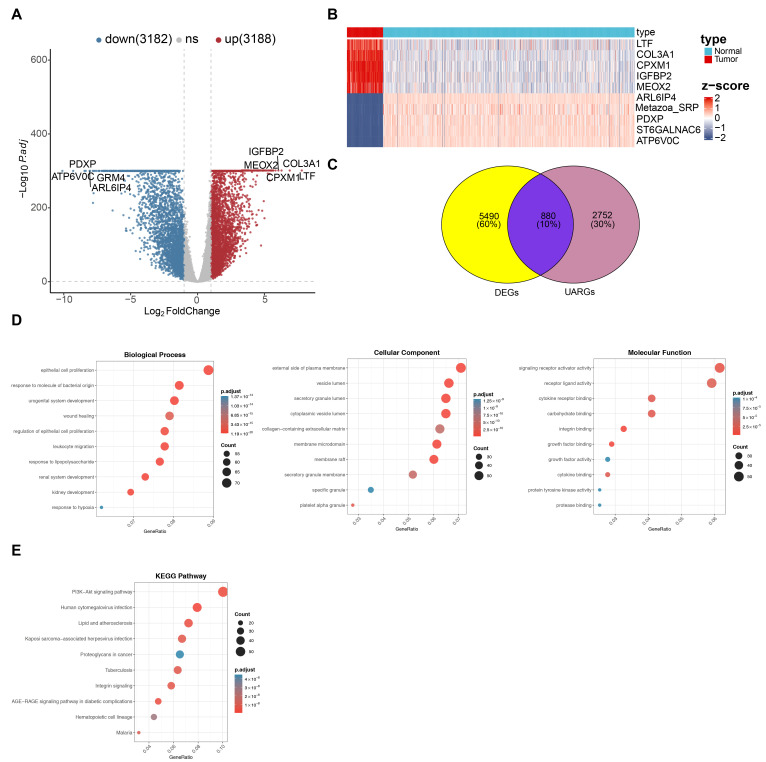
Identification and functional enrichment of candidate genes. (**A**) Volcano plot of the distribution of differentially expressed uric acid-associated genes (UARGs) between GBM patients and the control group; (**B**) heatmap of the expression levels of differentially expressed uric acid-related genes (UARGs) between GBM and control groups; (**C**) Venn diagram of candidate genes identified from differentially expressed UARGs in GBM and preset uric acid-associated genes; (**D**) GO enrichment analysis of the gene set obtained by intersection; and (**E**) KEGG enrichment analysis of the gene set obtained by intersection.

The TCGA-GBM and training set 2 identified 6310 DEGs, including 3188 up-regulated genes and 3182 down-regulated genes in the GBM group. The volcano plot illustrated the top five regulated DEGs (Figure 1A). In the heat map, these DEGs showed clearly separated expression patterns between GBM and normal specimens (Figure 1B). Overlap analysis of the 6310 DEGs with the 3632 UARGs resulted in 880 DE-UARGs (Figure 1C, Table S2). Subsequently, GO and KEGG enrichment analyses were performed on the 880 DE-UARGs to explore their potential molecular processes. A total of 2437 GO terms were enriched. Among the 2200 BP entries, “epithelial cell proliferation” and “response to lipopolysaccharide” were notably enriched. The 95 entries of the CC category included “external side of plasma membrane” and “vesicle lumen”. A total of 142 entries of the MF category suggested that DE-UARGs were closely associated with “receptor–ligand activity” and “growth factor binding” (adj. *p* < 0.05, Figure 1D, Table S3). Furthermore, KEGG analysis indicated that DE-UARGs were enriched in 118 KEGG pathways, including “PI3K-Akt signaling pathway”, “human cytomegalovirus infection”, and “AGE-RAGE signaling pathway in diabetic complications” (adj. *p* < 0.05, Figure 1E, Table S4). PPI mapping of the 880 DE-UARGs revealed a network consisting of 598 nodes and 1308 edges. In this topology, STAT3, MYC, TP53, CDK1, STAT4, and IL1B were identified as hub nodes with relatively high connectivity (Figure S1).

### 3.2. Construction and Validation of the UARG-Related Risk Model

**Figure 2:**
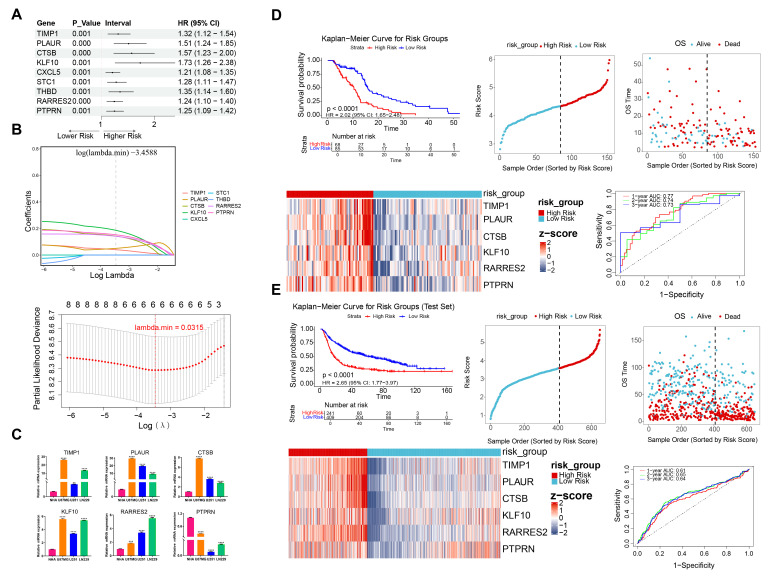
Establishment of a risk stratification model based on prognostic signatures. (**A**) Forest plot of univariate Cox regression analysis for prognostic genes. (**B**) LASSO regression coefficient path diagram and cross-validation curve of LASSO regression for prognostic gene selection. (**C**) RT-qPCR results of 6 key genes expression in NHA and human glioblastoma cell lines. TIMP1, PLAUR, CTSB, KLF10, RARRES2, and PTPRN. Multiple groups used one-way ANOVA test and Tukey’s post hoc test. Significance was set at *p* < 0.05 (** *p* < 0.01, *** *p* < 0.001, and **** *p* < 0.0001). (**D**,**E**) Risk score curves, survival status, expression heatmap, Kaplan–Meier curves, and ROC curves for prognostic model in TCGA cohort and CGGA cohort.

Univariate Cox regression analysis (HR ≠ 1, *p* < 0.001) and PH assumption tests (*p* > 0.05) identified nine prognostic UARGs whose expression was significantly associated with overall survival in TCGA-GBM. All nine candidate prognostic UARGs, including TIMP1, PLAUR, CTSB, KLF10, RARRES2, and PTPRN, were identified as potential risk factors (HR > 1) (Figure 2A and Figure S2). To optimize gene selection and improve model reliability, six prognostic UARGs (TIMP1, PLAUR, CTSB, KLF10, RARRES2, and PTPRN) were selected using LASSO regression analysis (lambda.min = 0.0315) (Figure 2B). Additionally, the expression levels of 6 prognostic UARGs were verified by RT-qPCR. The expression of TIMP1, PLAUR, CTSB, KLF10, and RARRES2 in human glioblastoma cell lines was significantly higher than in NHA cells, and the expression of PTPRN showed the opposite trend (*p* < 0.01) (Figure 2C).

Subsequently, the risk score equation for the GBM patients’ risk model was as follows: risk score = (0.040) × TIMP1 expression + (0.050) × PLAUR expression + (0.143) × CTSB expression + (0.186) × KLF10 expression + (0.141) × RARRES2 expression + (0.153) × PTPRN expression. Furthermore, using a risk score threshold of 38.66016 (high-/low-risk patients = 68/85), TCGA-GBM samples were divided into high-risk (*n* = 68) and low-risk (*n* = 65) groups to evaluate the six-UARG signature. In the HRG, higher risk scores were associated with shorter survival times and a gradual increase in the proportion of deaths. Expression levels of TIMP1, PLAUR, CTSB, KLF10, RARRES2, and PTPRN were relatively higher in the HRG. KM plots showed a notable survival disadvantage in the high-risk cohort, which was consistent with a relatively adverse prognosis for these GBM cases (*p* < 0.0001). The signature showed favorable OS prediction performance in GBM, with AUCs of 0.77, 0.74 and 0.73 at 1-, 2- and 3-year horizons, respectively (Figure 2D). Additionally, the consistent performance of the signature was verified in the CGGA validation cohort, which was similar to the outcomes observed in TCGA-GBM (Figure 2E).

Multidimensional comparative validation in this study demonstrated that the six-prognostic UARG signature was superior to the cuproptosis-related five-prognostic gene model [PMID: 36814929] in predictive performance in both the TCGA-GBM and CGGA independent cohorts. In the TCGA cohort, the hazard ratio of the UARG model (HR = 3.24, 95% CI: 2.15–4.89) was significantly higher than that of the cuproptosis-related model (HR = 2.29, 95% CI: 1.67–3.15 (Figure S3A). The areas under the time-dependent ROC curves (AUC) at 1, 2, and 3 years were all higher for the UARG model than for the control model, indicating that the UARG model achieved better risk stratification and long-term prognostic prediction (Figure S3B). Subgroup analyses revealed that the UARG model enabled stable patient risk stratification in patients aged ≤60 years, aged >60 years, and both genders, whereas the cuproptosis-related model showed limitations in several subgroups (Figure S3C). Collectively, the UARG prognostic signature was established as a more accurate and reliable tool for prognostic evaluation in patients with GBM, highlighting its significant clinical application value.

### 3.3. Risk-Stratified Immune-Infiltration Landscape in GBM

**Figure 3:**
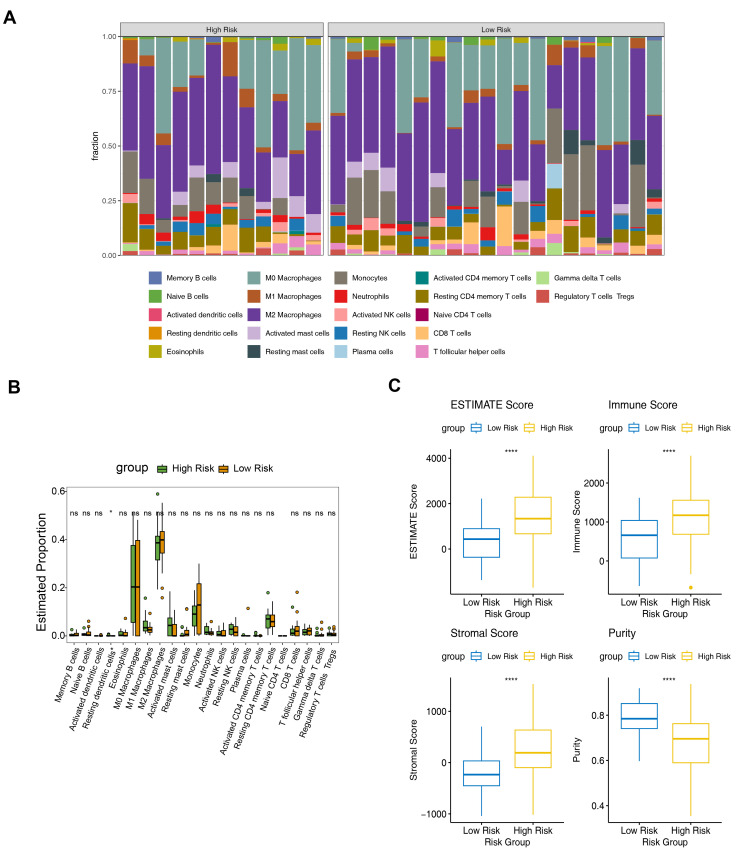
Correlation between uric acid-related prognostic genes and immune cell infiltration levels. (**A**) Infiltration proportions of 22 types of immune cells in the high-risk and low-risk groups. (**B**) Infiltration status of 22 types of immune cells in the high-risk and low-risk groups. The *x*-axis represents different cell types, the *y*-axis represents the cell proportion in each sample, and cell types with significant differences are marked with asterisks (* *p* < 0.05), “ns” indicates not significant (*p* > 0.05). Green dots indicate the immune cell infiltration proportion per sample in the high-risk group, while orange dots indicate that in the low-risk group. (**C**) ESTIMATE algorithm analysis in the high-risk and low-risk groups. (**** *p* < 0.0001). A yellow dot represents an original sample data point. Although a point appearing below the Immune Score can be statistically considered an outlier, it reflects true biological variation.

Tumor-infiltrating leukocytes may significantly shape GBM trajectory and correlate with patient prognosis. Immune cell abundance was compared between high- and low-risk TCGA-GBM groups (Figure 3A). The HRG showed a significant increase in resting dendritic cell infiltration (*p* < 0.05, Figure 3B). These findings suggested that uric acid-associated abnormal immune infiltration in GBM may have important clinical significance. ESTIMATE-derived immune, stromal, and ESTIMATE scores were significantly higher in the high-risk group, while tumor purity was significantly decreased (all *p* < 0.0001) (Figure 3C). This finding suggested that the aggressive phenotype of high-risk GBM is closely associated with a TME characterized by abundant stromal components and immune cell infiltration, which may serve as a physical and biological barrier contributing to treatment resistance.

### 3.4. UARG-Derived Signature as a Pharmacogenomic Predictor of Therapeutic Responsiveness in GBM

**Figure 4:**
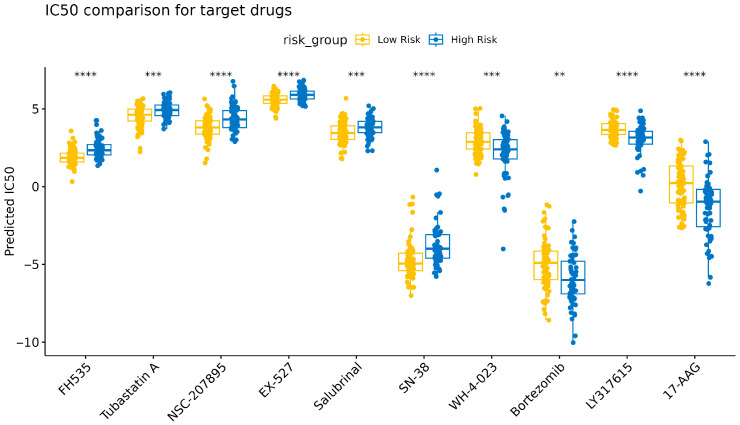
Drug sensitivity analyses of anticancer drugs in the high-risk and low-risk groups. The *x*-axis represents different drugs, and the *y*-axis represents IC_50_ values. The Wilcoxon test was used to determine statistical significance (** *p* < 0.01, *** *p* < 0.001, **** *p* < 0.0001).

Systematic pharmacogenomic profiling revealed significant differential sensitivity to 56 anticancer agents (Figure S4). The 10 agents showing the most prominent risk-stratified sensitivity were selected for further analysis. The LRG exhibited significantly higher sensitivity to FH535, tubastatin A, NSC-207895, EX-527, and SN-38 (*p* < 0.01), which suggested that the LRG may be more responsive to these agents. In contrast, significantly lower IC_50_ values were observed in the HRG for WH-4-023, bortezomib, LY317615, and 17-AAG (*p* < 0.01), indicating increased chemosensitivity in this subgroup (Figure 4, Table S5).

### 3.5. Mutational-Landscape Comparison Between High-Risk and Low-Risk Subgroups

**Figure 5:**
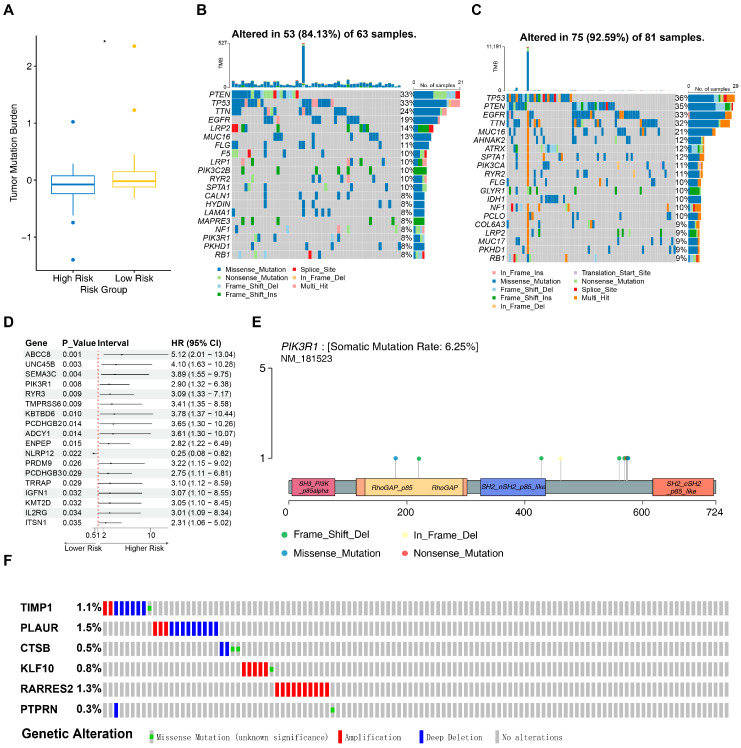
Tumor mutation analysis in high-risk and low-risk groups. (**A**) Differences in tumor mutation burden (TMB) scores between high-risk and low-risk groups (* *p* < 0.05); Both yellow and blue dots denote the raw sample data points, showing the distribution density, dispersion, and potential outliers within each group. (**B**) Waterfall plot of mutation analysis in the high-risk group. The *x*-axis represents different samples, the *y*-axis represents mutated genes, and the color of each cell indicates the type of mutation. (**C**) Waterfall plot of mutation analysis in the low-risk group. (**D**) Forest plot of univariate Cox regression analysis showing the prognostic hazard ratios (HRs) of differentially mutated genes. Genes with HR > 1 (on the right) are defined as risk genes, while those with HR < 1 (on the left) are protective genes. (**E**) Lollipop plot of protein mutation sites of the key mutated gene PIK3R1. (**F**) Distribution of point mutations and copy number variations in common genes of glioma, analyzed using the merged glioblastoma (GBM) and low-grade glioma samples from the TCGA dataset provided by cBioportal (https://www.cbioportal.org, accessed on 3 April 2025).

Progressive accumulation of gene mutations is widely recognized as a key driver of tumorigenesis and malignant evolution [39]. Accordingly, mutation analysis revealed that significantly higher TMB scores were observed in the LRG (*p* < 0.05, Figure 5A). Somatic mutation data may contribute to a better understanding of factors associated with distinct clinical outcomes. Mutational profiling of TCGA-GBM showed that missense variants represented the most common type of alteration. PTEN and TP53 were the most frequently mutated genes in the HRG (each 33%), followed by TTN, EGFR, LRP2, MUC16, FLG, F5, LRP1 and PIK3C2B (Figure 5B). In the LRG, TP53 (36%) and PTEN (35%) were the most common mutations, followed by EGFR, TTN, MUC16, AHNAK2, ATRX, SPTA1, PIK3CA and RYR2 (Figure 5C). Notably, PTEN, TP53, TTN and EGFR were among the top four mutated genes in both groups, suggesting their potentially important roles in GBM progression. Subsequently, differential mutation analysis between the two risk groups identified 207 DMGs. Univariate Cox regression analysis and PH assumption tests further identified 18 key mutated genes (Figure 5D, Table S6). Mutation analysis demonstrated that PIK3R1 had a mutation rate of 6.25% in GBM, which was relatively high in the TCGA-GBM cohort (Figure 5E). Lollipop plots illustrating mutation sites of other key mutated genes are shown in Figure S5.

To investigate the mutational profiles of prognostic UARGs, data from GBM patients in the cBioPortal database were analyzed. Amplification and deep deletion were identified as common variant types. Amplification was predominantly observed in TIMP1, PLAUR, KLF10, and RARRES2, suggesting that overexpression of these genes might be associated with GBM progression. Deep deletion was mainly detected in CTSB, implying that loss of function of this gene might contribute to tumorigenesis (Figure 5F).

### 3.6. RARRES2 and PTPRN as Independent Prognostic Factors in GBM

**Figure 6:**
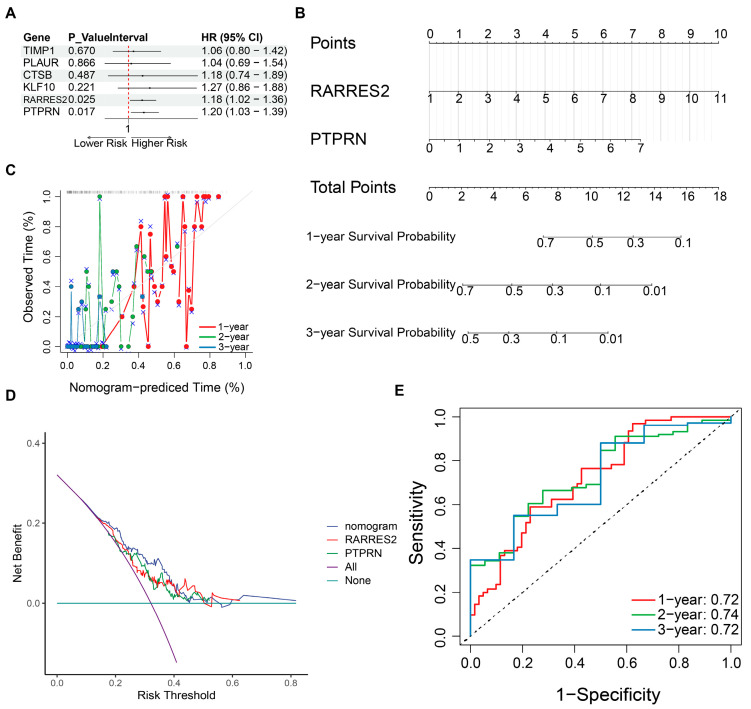
Independent prognostic analysis of candidate genes in the glioma cohort. (**A**) multivariate Cox regression analysis for independent prognostic factors; (**B**) Nomogram analysis for the prognosis of two key genesc (RARRES2 and PTPRN) in glioma patients; (**C**) Calibration curves for 1-, 2-, and 3-year survival of the nomogram model; The gray dashed diagonal line represents the ideal reference line, where predicted probabilities perfectly match observed outcomes. Colored dots indicate the predicted survival probabilities from the nomogram (e.g., red for 1-year, green for 2-year, blue for 3-year). Blue cross symbols with gray error bars (±1 SD) represent the actual observed survival probabilities. The closer the colored dots are to the diagonal line, the better the predictive accuracy of the model. (**D**) Decision curves for 1-, 2-, and 3-year survival of the gene signature-based model; (**E**) ROC curves for 1-, 2-, and 3-year survival of the gene signature-based model. (The dashed line represents the diagonal line, which serves as the reference line for an AUC of 0.5).

Multivariate Cox regression analysis with verification of the proportional hazards assumption identified RARRES2 and PTPRN as independent prognostic factors in TCGA-GBM (Figure 6A and Figure S6). Subsequently, a nomogram incorporating RARRES2 and PTPRN was established to predict 1-, 2- and 3-year survival probabilities and evaluate its clinical utility in GBM (Figure 6B). Higher total points were associated with shorter survival in patients with GBM. Notably, calibration curves showed good consistency between the predicted and observed survival probabilities, supporting the favorable predictive performance of this model (Figure 6C). Decision curve analysis indicated that the net benefit of the nomogram was better than that of individual variables (Figure 6D). ROC analysis demonstrated acceptable discriminative ability, with AUC values of 0.72, 0.74 and 0.72 for 1-, 2- and 3-year survival, respectively (Figure 6E). In summary, the UARG-based nomogram may serve as a useful tool to predict the prognosis of GBM patients and provide a reference for clinical decision-making.

### 3.7. Panoramic Single-Cell Atlas of GBM

**Figure 7:**
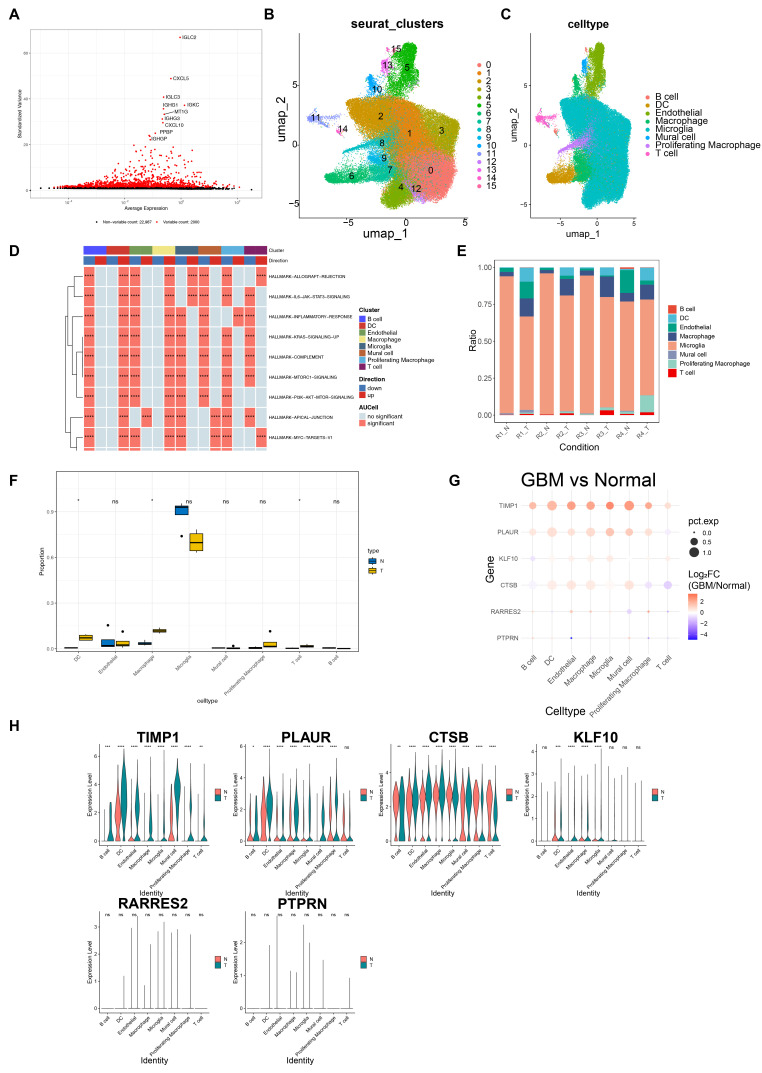
GBM data were downloaded from the public database GSE162631 for single-cell sequencing analysis. (**A**) Gene variability plot, where the *x*-axis represents the mean value of gene expression, the *y*-axis represents the normalized variance, and larger values indicate higher gene variability; (**B**) UMAP plot of cell cluster classification, where the *x*-axis and *y*-axis represent UMAP_1 and UMAP_2 respectively, and the dots are colored according to different cell clusters; (**C**) cell annotation plot of GBM single-cell sequencing data; (**D**) enrichment analysis of annotated cell types, where the *x*-axis represents different cell types and the *y*-axis represents each pathway (**** *p* < 0.0001); (**E**) proportion of each cell cluster type in the GBM group; (**F**) comparison of differential cells between GBM tissue samples and normal tissue samples (* *p* < 0.05); The small black dots represent the proportion values of that cell type in each sample; (**G**) expression differences in prognostic genes between GBM patient tissue samples and normal control samples; and (**H**) expression of 6 prognostic genes in different cell types of GBM(* *p* < 0.05, ** *p* < 0.01, *** *p* < 0.001, **** *p* < 0.0001).

Post-integration filtering and quality control reduced the GBM dataset from 120,279 cells and 33,538 genes to 97,382 cells and 24,987 genes (Figure S7A). Then, the top 2000 highly variable genes were retained for subsequent analysis (Figure 7A). PCA combined with a scree plot was used to select the top 25 principal components for downstream analysis (*p* < 0.05, Figure S7B–D). Cells from different samples were integrated, and batch effects were mitigated, followed by UMAP clustering (Figure S7E,F). Subsequently, cells were divided into 16 distinct clusters (Figure 7B). Thereafter, all cells were annotated into eight cell types: microglia, endothelial cells, B cells, T cells, macrophages, proliferating macrophages, dendritic cells (DCs), and mural cells (Figure 7C). A bubble plot was generated to show the expression patterns of marker genes (Figure S7G, Table S7). Furthermore, a total of 400 significant pathways, including “interleukin-33 signaling”, “IL6-JAK-STAT3 signaling”, “PI3K-AKT-mTOR signaling”, “apical junction”, “complement”, and “allograft rejection”, were enriched in the annotated cells (Figure 7D, Table S8). The histogram displayed the relative proportion of the eight annotated cell types, with microglia as the most abundant population in the single-cell dataset (Figure 7E). The abundance of DCs, macrophages, and T cells was significantly different between GBM and adjacent non-tumor tissues (*p* < 0.05, Figure 7F). TIMP1 and PLAUR exhibited high expression levels in all cell types (Figure 7G). Notably, expression levels of TIMP1 and CTSB in DCs, macrophages, and T cells were significantly different between the two groups (*p* < 0.01, Figure 7H). Therefore, DCs, macrophages, and T cells were identified as key cell populations for subsequent analysis.

### 3.8. Cell Communication and Pseudo-Time Landscapes in Key Cells

**Figure 8:**
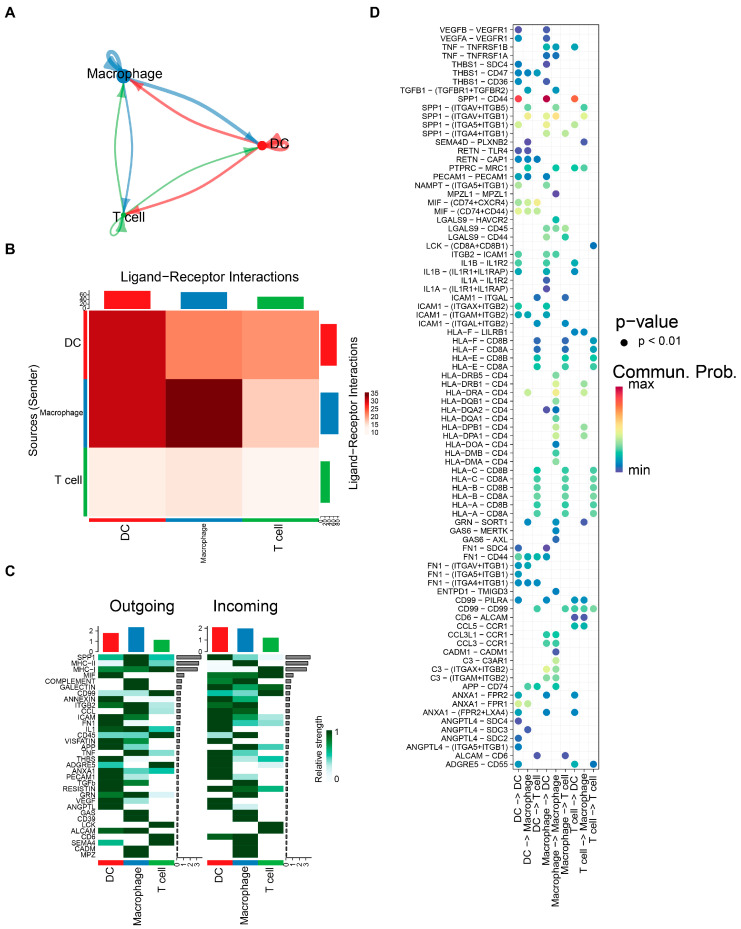
Cell communication analysis in DCs, macrophages, and T cells. (**A**) Interaction network plot of DCs, macrophages, and T cells in GBM microenvironment; Colored arrows indicate the direction of communication from the signal-sending cell (source) to the signal-receiving cell (target); (**B**) predicted interaction heatmap between signal-sending cells and signal-receiving cells; (**C**) signal transduction pathway heatmap of cell–cell communication; (**D**) ligand–receptor interaction bubble plot. The *x*-axis represents cell types (with the cell types on the left side of the arrow as signal-sending cells and those on the right side as signal-receiving cells), the *y*-axis represents significantly interacting ligand–receptor pairs, and the color indicates communication probability.

Following the identification of DCs, macrophages, and T cells as key cells, a cell communication network diagram was generated to illustrate the number and strength of interactions among these key cell populations. Notably, DCs and macrophages showed relatively strong reciprocal interactions across most tissue specimens (Figure 8A,B). Subsequently, all predicted incoming and outgoing signaling patterns in DCs, macrophages, and T cells were analyzed. In the signaling patterns of DCs, IL1 and VEGF showed relatively high intensity; in macrophages, MHC-II and ITGB2 exhibited relatively high intensity; and in T cells, LCK and CD99 displayed relatively high intensity (Figure 8C). Intercellular wiring among key populations was mainly mediated by the SPP1-CD44 and SPP1-(ITGAV+ITGB1) receptor–ligand axes (Figure 8D).

**Figure 9:**
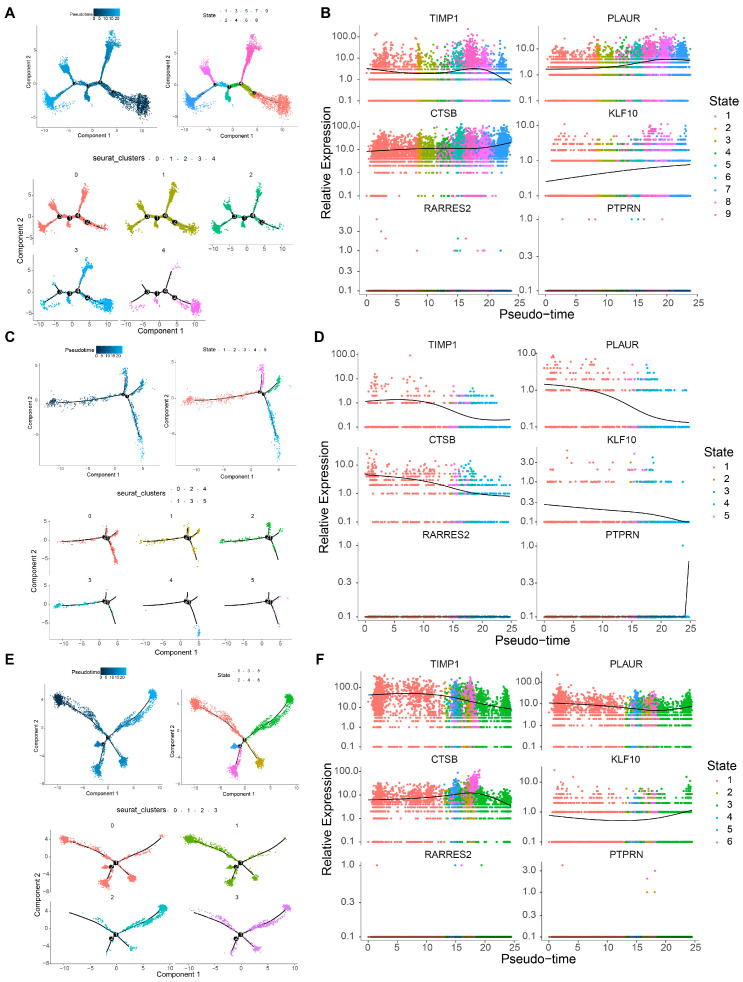
Pseudo-time landscapes in macrophages, dendritic cells, and T cells. (**A**) Pseudotime differentiation trajectory plot of cell states calculated by Monocle, and pseudotime differentiation trajectory of macrophages; Different numbers represent cell cluster IDs. The black lines (including the main trajectory and branches) represent the cell differentiation trajectory. The branch points in the trajectory indicate where cells can differentiate along distinct directions toward different cell fates; (**B**) the expression levels of TIMP1, PLAUR, CTSB, KLF10, RARRES2, and PTPRN in macrophages; (**C**) pseudotime differentiation trajectory plot of cell states calculated by Monocle, and pseudotime differentiation trajectory of dendritic cells; (**D**) the expression levels of TIMP1, PLAUR, CTSB, KLF10, RARRES2, and PTPRN in dendritic cells; (**E**) pseudotime differentiation trajectory plot of cell states calculated by Monocle, and pseudotime differentiation trajectory of T cells; (**F**) the expression levels of TIMP1, PLAUR, CTSB, KLF10, RARRES2, and PTPRN in T cells.

Differentiation trajectories of macrophages were resolved by pseudo-time analysis, and the cells were partitioned into five subgroups and nine states (Figure S8A and Figure 9A). State 9 appeared to be the earliest branch, while state 7 seemed to be terminally differentiated; concurrently, the expression dynamics of prognostic UARGs were mapped across the macrophage pseudo-time trajectory. Along the macrophage differentiation trajectory, PLAUR, CTSB, and KLF10 showed a monotonic increase in expression. The expression levels of TIMP1 exhibited a pattern of initial stability followed by a decrease. For RARRES2 and PTPRN, their expression levels were nearly undetectable (Figure 9B). Pseudo-time analysis was performed to infer the differentiation trajectory of T cells, which were clustered into six subgroups and five states (Figure S8B and Figure 9C). Notably, states 3 and 4 appeared to be at an earlier differentiation stage, while state 1 showed a relatively later differentiation pattern. Along the T-cell pseudo-time trajectory, TIMP1, PLAUR, CTSB, and KLF10 displayed a monotonic decline in expression. PTPRN expression was initially nearly undetectable but increased sharply at the terminal stage of differentiation. For RARRES2, its expression levels remained almost undetectable throughout the trajectory (Figure 9D). DCs were clustered into four subgroups and six states (Figure S8C and Figure 9E). Notably, state 3 appeared to be at an earlier differentiation stage, while state 1 showed a relatively later differentiation pattern. As DCs differentiated, the overall expression levels of TIMP1, PLAUR, and CTSB remained stably high, whereas KLF10 maintained stably low expression. The expression of RARRES2 and PTPRN was almost undetectable (Figure 9F). The secondary clustering process of the above key cells was shown in Figures S9–S11.

## 4. Discussion

GBM is the most lethal primary intracranial tumor. It not only disrupts the blood-brain tumor barrier but also exhibits remarkable intrinsic plasticity, allowing it to reversibly adapt to the dynamic tumor microenvironment and generate tumor heterogeneity [40,41,42]. In addition, hypoxia and hypoglycemia can deeply activate the transcription of the MIF gene, significantly upregulate MIF mRNA expression and increase extracellular MIF protein levels, which may in turn regulate neovascularization in gliomas [43,44,45]. Notably, uric acid exerts a biphasic effect on the blood–brain barrier: it protects the blood–brain barrier at physiological concentrations but impairs it when abnormally elevated [46]. As the final product of purine metabolism, uric acid is also a potent immune–inflammatory regulator and peroxynitrite scavenger, occupying a central role in the pathobiology of tumors. Both observational and mechanistic studies have shown that elevated serum uric acid levels are positively correlated with tumor burden in patients, and intratumoral uric acid accumulation can promote peroxynitrite-mediated cell proliferation [47,48]. Although some understanding of the above characteristics of glioblastoma has been achieved, radical treatment remains difficult even with maximized multimodal therapy. This dilemma has shifted research focus toward its dysregulated redox and metabolic regulatory networks [47,48]. However, the prognostic value of uric acid-related genes in glioblastoma remains unclear. Accordingly, in the present study, bulk transcriptomic and single-cell transcriptomic datasets were integrated to construct a uric acid-related gene signature (UARG signature) consisting of six genes, which could effectively stratify glioblastoma patients into distinct risk subgroups. Comprehensive analyses of the immune microenvironment, drug sensitivity profiles, and mutation landscapes revealed that different risk groups possessed unique biological characteristics, providing a theoretical basis for individualized therapeutic strategies. The six-gene signature identified in this study includes TIMP1, PLAUR, CTSB, KLF10, RARRES2, and PTPRN. These genes are collectively involved in the regulation of extracellular matrix remodeling, immune microenvironment modulation, and tumor cell signaling. The biological significance of these genes and their impact on the prognosis of glioblastoma are discussed in the following sections.

Tissue inhibitor of metalloproteinases 1 (TIMP1) is a secreted glycoprotein that blocks matrix metalloproteinase activity. In GBM, NF-κB directly binds the TIMP1 promoter to drive transcription, thereby accelerating tumor-cell proliferation and overall tumor growth [49]. When bevacizumab (a VEGF-neutralizing antibody) is combined with topoisomerase-1 (TOP1) inhibitors as second-line therapy, TIMP1 over-expression was found to blunt the cytotoxic effect of TOP1 inhibitors, indicating that TIMP1 abundance predicts chemotherapeutic resistance in GBM patients [50,51]. Complementing TIMP1’s role in matrix remodeling, the plasminogen activator/urokinase receptor (PLAUR) specifies the urokinase-type plasminogen activator receptor, a GPI-anchored receptor that sequesters pro- and active urokinase at the plasma membrane, thereby localizing plasmin formation and driving pericellular matrix proteolysis [52]. Single-cell and bulk RNA analyses have positioned PLAUR at the hub of a pro-tumor microenvironment that is both hypoxic and immunosuppressive; its expression drives mesenchymal transition, the most aggressive and therapy-refractory GBM subtype [53]. High PLAUR expression is an independent prognostic marker: such tumors contain fewer CD8^+^ T cells, more M2-polarized macrophages, and strong positive correlations with multiple immunosuppressive signatures [54]. Cathepsin B (CTSB) is a lysosomal cysteine endo- and exo-peptidase that orchestrates intracellular protein turnover [55]. In GBM, CTSB is released extracellularly, where it degrades laminin, collagen IV, and fibronectin, thereby clearing physical barriers for tumor-cell migration and invasion [56]. Consistently, CTSB overexpression correlates with higher WHO grade, increased motility in vitro, and more extensive intracranial dissemination in pre-clinical models. Krüppel-like factor 10 (KLF10) is a zinc-finger transcription factor widely recognized as a tumor suppressor [57]. In pancreatic cancer, step-wise loss of KLF10 parallels disease progression, making it a staging biomarker [58]. Recent GBM cohort analyses reveal that low KLF10 expression is associated with shorter overall survival, underscoring its prognostic value [59]. Retinoic-acid-receptor-responder-2 (RARRES2) was originally described as a tumor suppressor that inhibits p38 signaling and cell invasion in adrenocortical carcinoma [60] and that remodels the microenvironment to restrain breast cancer growth [61]. Conversely, bioinformatic dissection of 1087 gliomas showed that elevated RARRES2 mRNA predicts worse clinical outcome in both LGG and GBM, and siRNA-mediated silencing impairs tumor-cell viability, suggesting that glioma cells have co-opted RARRES2 for oncogenic support [62]. This context-dependent functional switch may reflect distinct signaling networks or microenvironmental cues in the central nervous system that reprogram RARRES2 from a tumor suppressor to a pro-survival factor. Protein-tyrosine-phosphatase receptor-type N (PTPRN) is a type-I transmembrane protein with a catalytically inactive PTP domain that participates in neuro-endocrine vesicle traffic. In malignant gliomas, elevated PTPRN serves as an autonomous predictor of adverse prognosis and shows a significant negative correlation with overall survival [63]. Functionally, PTPRN scaffolds NEDD4L to the NaV1.2 sodium channel, promoting channel ubiquitination and internalization; CRISPR deletion of PTPRN increases NaV1.2 current and neuronal excitability, whereas over-expression dampens it [64]. In parallel, PTPRN binds HSP90AA1 to activate PI3K/AKT signaling, enhancing proliferation, epithelial-to-mesenchymal transition and invasion [65]. These data implicate PTPRN as a dual regulator of electrical and oncogenic signaling in GBM. In this study, the expression levels of TIMP1, PLAUR, CTSB, KLF10, and RARRES2 were further verified to be significantly higher in human glioblastoma cell lines than in NHA cells, whereas the expression of PTPRN exhibited the opposite trend. Collectively, TIMP1, PLAUR, CTSB, KLF10, RARRES2 and PTPRN constitute a functionally integrated panel that captures key hallmarks of GBM pathobiology, including extracellular matrix remodeling, immune evasion, metabolic reprogramming, and therapeutic resistance. The differential expression patterns of these six genes between GBM cell lines and normal astrocyte cells, as verified in this study, further support their functional relevance in GBM pathogenesis, thereby providing a comprehensive framework for risk stratification and a basis for exploring potential therapeutic targets.

To further explore the molecular mechanisms mediated by UARGs, we performed signaling pathway enrichment analysis on differentially expressed UARGs. The results showed that the PI3K-Akt and AGE-RAGE signaling pathways were the most significantly enriched pathways, both of which are crucial for GBM development and progression. Notably, resting dendritic cells are closely related to the immune microenvironment of GBM and represent an important immune feature of GBM [66]. Significant differences in immune score, stromal score, and ESTIMATE score exist between HRG and LRG in immune-based risk models [67]. In our UARG-based risk model, the HRG exhibited markedly increased resting dendritic cell infiltration, elevated immune, stromal, and ESTIMATE scores, and decreased tumor purity compared with the LRG, suggesting that UARGs may contribute to GBM progression by regulating the tumor immune microenvironment. The PI3K/AKT pathway is constitutively hyperactivated and promotes tumorigenesis, proliferation, invasion, and metastasis [68]. Its downstream NF-κB enhances cancer cell survival and fosters a pro-inflammatory microenvironment [69]. Human cytomegalovirus infection activates PI3K/AKT, NF-κB, and mTOR to inhibit apoptosis and accelerate proliferation in GBM cells [70]. The AGE-RAGE axis chronically activates the same cascades, conferring proliferation, anti-apoptosis, and chemoresistance [71] and promoting angiogenesis, mesenchymal transition, and stemness in GBM [72]. Therefore, crosstalk between the PI3K-Akt and AGE-RAGE pathways allows UARGs to coordinately regulate GBM progression and therapeutic response, partly by modulating the immune microenvironment via pro-inflammatory effects.

Beyond transcriptional alterations, genomic mutations represent another critical layer of molecular heterogeneity in GBM. Somatic-mutation profiles from TCGA were compared between high- and low-risk groups to delineate differentially altered genes. and found out that mutant genes such as PTEN, TP53, TTN, and EGFR in the high-risk population are key drivers of GBM progression [73,74,75]. In addition, drug resistance in GBM patients poses a major therapeutic challenge, and predicting drug sensitivity enables personalized treatment selection by accounting for individual differences in responses to chemotherapeutic agents. Therefore, our findings provide multiple drug therapy options for GBM patients (WH-4-023, bortezomib, LY317615, and 17-AAG exhibit higher sensitivity in the high-risk group), although the final efficacy needs to be verified through clinical trials. In addition, drug resistance in GBM patients poses a major therapeutic challenge. Our drug sensitivity analysis identified several compounds, including bortezomib, WH-4-023, LY317615, and 17-AAG, that exhibited differential sensitivity between high- and low-risk groups. It is noteworthy that patients in the high-risk group showed higher predicted sensitivity to bortezomib, a proteasome inhibitor that can overcome resistance to alkylating agents by suppressing MGMT expression [76,77,78]. These findings suggest that UARG-based risk stratification may guide personalized drug selection, though clinical validation is required.

Single-cell analysis revealed that dendritic cells, macrophages, and T cells were key cellular components expressing our prognostic UARGs in the GBM microenvironment [79,80,81]. Cell communication analysis demonstrated strong interactions between DCs and macrophages, mediated by SPP1-CD44 and SPP1-ITGAV+ITGB1 receptor–ligand pairs, suggesting coordinated immune regulation. Pseudotime trajectory analysis showed that expression of TIMP1, PLAUR, and CTSB dynamically changed during macrophage and T cell differentiation, indicating that these UARGs may influence immune cell maturation states and thereby shape the immunosuppressive microenvironment in GBM [82,83,84].

In summary, this study takes uric acid as the entry point to comprehensively elucidate the molecular mechanisms of prognosis-related UARGs in GBM. The derived risk signature maintained consistent accuracy across independent cohorts and displayed strong predictive power for patient outcomes, underscoring its translational potential. Moreover, high-resolution pharmacogenomic screening unveils actionable targets for immunotherapy and next-generation drug design, whereas single-cell interrogation offers a refined view of GBM pathobiology.

Despite its strengths, this study has several limitations. First, it relied solely on bioinformatic analyses of public databases (TCGA and CGGA), representing a purely in silico study. Lacking experimental validation, such as IHC, the results have not been verified at the tissue level. Meanwhile, as a retrospective predictive analysis, the associated molecular mechanisms and clinical value of the findings require further functional validation using cellular models, animal experiments, and clinical cohorts. Second, although this study provides a theoretical framework for further clarifying the factors influencing the prognosis of GBM patients, additional samples from diverse GBM cohorts and multi-level experiments are still needed to validate the predictive efficacy of the screened uric acid-associated prognostic genes. Furthermore, further research is required to investigate the crosstalk between these UARGs and other metabolic pathways, as well as signal transduction networks operative in GBM. Concurrently, single-cell sequencing datasets can be further explored to elucidate the differentiation processes of key cell types, their roles in GBM, and the molecular mechanisms governing cell differentiation. In future studies, we will collect clinical samples and establish cellular models to perform experimental validation (including RT-qPCR and IHC) for core prognostic genes. These efforts will facilitate an in-depth exploration of their molecular mechanisms and clinical significance, thereby enhancing the reliability and clinical applicability of the study results.

## 5. Conclusions

By integrating bulk and single-cell transcriptomes, we evaluated the prognostic significance of uric acid-related genes in GBM progression. We developed and validated a six-gene prognostic signature (TIMP1, PLAUR, CTSB, KLF10, RARRES2, and PTPRN) that may help stratify GBM patients into subgroups with distinct survival outcomes. Further characterization suggested that high-risk patients were associated with increased resting dendritic cell infiltration, differential drug sensitivity patterns, and distinct mutational profiles, particularly involving PTEN and TP53. Single-cell analysis indicated that dendritic cells, macrophages, and T cells were among the key cellular sources of the prognostic UARGs, with active intercellular communication networks potentially mediated by SPP1-CD44 and SPP1-ITGAV+ITGB1 signaling pathways. A nomogram incorporating RARRES2 and PTPRN showed favorable predictive performance for clinical outcomes. Collectively, these findings provide insights into UARG-associated pathways in glioblastoma pathobiology and offer potential biomarkers that may support risk stratification and inform precision therapeutic strategies.

## Data Availability

Transcriptome data of glioblastoma multiforme (GBM) patients were sourced from The Cancer Genome Atlas (TCGA) database (http://cancergenome.nih.gov/, accessed on 3 April 2025). The GTEx database (https://www.gtexportal.org/, accessed on 3 April 2025) was employed to download 1148 normal brain tissue samples. The externally validated transcriptome analyses and clinical information of GBM patients were sourced from the Chinese Glioma Genome Atlas (CGGA) data portal (http://www.cgga.org.cn/, accessed on 3 April 2025). Transcriptome data from the single-cell RNA sequencing (scRNA-seq) dataset GSE162631 associated with GBM were retrieved from the Gene Expression Omnibus (GEO) database (http://www.ncbi.nlm.nih.gov/geo/, accessed on 3 April 2025). GSE162631 (platform: GPL24676) included four GBM tumor tissue samples and four peritumoral tissues. Additionally, a total of 3632 uric acid-related genes (UARGs) (PMID: 38213580) (Table S1) were sourced from the Genecards database (https://www.genecards.org/, accessed on 3 April 2025). The drug sensitivity analysis was conducted with the Genomics of Drug Sensitivity in Cancer (GDSC) database (https://www.cancerrxgene.org/, accessed on 3 April 2025) to provide management recommendations for GBM. For the mutation status of prognostic UARGs, a mutation analysis was performed according to the GBM data from the cBioPortal database (https://www.cbioportal.org/, accessed on 3 April 2025). All PCR/qPCR data generated in this study are presented in the main text and Supplementary Tables.

## Supplementary Materials

The following supporting information can be downloaded at: https://www.mdpi.com/article/10.3390/cancers18081297/s1, Figure S1: Protein–protein interaction (PPI) analysis of the gene set obtained by intersection; Figure S2: Proportional hazards assumption test of nine candidate genes; Figure S3A: Kaplan–Meier survival analysis of UARG prognostic signature and cuproptosis-related prognostic model in TCGA and CGGA cohorts. Figure S3B: ROC curves for UARG prognostic signature and cuproptosis-related prognostic model in TCGA and CGGA cohorts. Figure S3C: Analysis of UARG model and cuproptosis model by gender, age, and HR in TCGA and CGGA cohorts. Figure S4: Drug sensitivity analyses of 56 anticancer drugs in the high-risk and low-risk groups; Figure S5: Lollipop plots depicting protein mutation sites for 17 key mutated genes; Figure S6: proportional hazards assumption test of TIMP1, PLAUR, CTSB, KLF10, RARRES2, and PTPRN; Figure S7A: Quality control of data from public database GSE162631 for single-cell sequencing analysis; Figure S7B: Dimensionality reduction result plot of principal component analysis (PCA); Figure S7C: PCA permutation test analysis; Figure S7D: PCA scree plot analysis; Figure S7E,F: UMAP plot of sample clustering before (Figure S7E) and after (Figure S7F); Figure S7G: Dot plot of marker genes for cell annotation batch effect removal; Figure S8A: UMAP plot of cell cluster classification in macrophages; Figure S8B: UMAP plot of cell cluster classification in dendritic cells; Figure S8C: UMAP plot of cell cluster classification in T cells; Figure S9: Secondary clustering process on macrophages; Figure S10: Secondary clustering process on T cells; Figure S11: Secondary clustering process on dendritic cells; Table S1: A total of 3632 UARGs downloaded from the Genecards database (https://www.genecards.org/, accessed on 3 April 2025); Table S2: The gene list of 880 genes obtained from the intersection of the two datasets; Table S3: Gene Ontology functional enrichment analysis data of the 880 genes; Table S4: KEGG functional enrichment analysis data of the 880 genes; Table S5: *p*-value for the top 10 difference drugs between HRS and LRG. Table S6: The bubble plot data of 18 key mutated genes; Table S7: Marker genes of eight annotated cells; Table S8: A total of 400 significant pathways’ enrichment analysis data from eight annotated cells.

## Abbreviations

The following abbreviations are used in this manuscript:

GBM	Glioblastoma Multiforme
TCGA	The Cancer Genome Atlas
UA	Uric Acid
UARGs	Uric Acid-Related Genes
GEO	Gene Expression Omnibus
ROC	Receiver Operating Characteristic
AUC	Area Under the Curve

## References

[1] Siegel R.L., Miller K.D., Wagle N.S., Jemal A. (2023). Cancer statistics, 2023. CA A Cancer J. Clin..

[2] Zhao T., Ge H., Lin C., Wu X., Chen J. (2025). Glycosylation Gene Signatures as Prognostic Biomarkers in Glioblastoma. Ann. Clin. Transl. Neurol..

[3] Kreatsoulas D., Bolyard C., Wu B.X., Cam H., Giglio P., Li Z. (2022). Translational landscape of glioblastoma immunotherapy for physicians: Guiding clinical practice with basic scientific evidence. J. Hematol. Oncol..

[4] Wang H.L., Li S., Ma C.C., Zheng X.H., Wu H.Y., Chang C.X., Yang Z.H., Wang J.W., Pan F.M., Zhao B. (2025). Characterization and prognostic of CD8 + TIM3 + CD101 + T cells in glioblastoma multiforme. Cell Biosci..

[5] Yeo A.T., Rawal S., Delcuze B., Christofides A., Atayde A., Strauss L., Balaj L., Rogers V.A., Uhlmann E.J., Varma H. (2022). Single-cell RNA sequencing reveals evolution of immune landscape during glioblastoma progression. Nat. Immunol..

[6] Hoggarth A.R., Muthukumar S., Thomas S.M., Crowley J., Kiser J., Witcher M.R. (2024). Clinical Theranostics in Recurrent Gliomas: A Review. Cancers.

[7] Ramos G.K., Goldfarb D.S. (2022). Update on Uric Acid and the Kidney. Curr. Rheumatol. Rep..

[8] Wang Q., Wen X., Kong J. (2020). Recent Progress on Uric Acid Detection: A Review. Crit. Rev. Anal. Chem..

[9] Kuwabara M., Ae R., Kosami K., Kanbay M., Andres-Hernando A., Hisatome I., Lanaspa M.A. (2025). Current updates and future perspectives in uric acid research, 2024. Hypertens. Res. Off. J. Jpn. Soc. Hypertens..

[10] Wu B., Zheng C., Mao C. (2025). Risk factors and a new nomogram for glioblastoma: Based on a retrospective study. Front. Immunol..

[11] Cabău G., Crișan T.O., Klück V., Popp R.A., Joosten L.A.B. (2020). Urate-induced immune programming: Consequences for gouty arthritis and hyperuricemia. Immunol. Rev..

[12] Liu N., Xu H., Sun Q., Yu X., Chen W., Wei H., Jiang J., Xu Y., Lu W. (2021). The Role of Oxidative Stress in Hyperuricemia and Xanthine Oxidoreductase (XOR) Inhibitors. Oxidative Med. Cell. Longev..

[13] Ma P., Amemiya H.M., He L.L., Gandhi S.J., Nicol R., Bhattacharyya R.P., Smillie C.S., Hung D.T. (2023). Bacterial droplet-based single-cell RNA-seq reveals antibiotic-associated heterogeneous cellular states. Cell.

[14] Guo S., Liu X., Zhang J., Huang Z., Ye P., Shi J., Stalin A., Wu C., Lu S., Zhang F. (2023). Integrated analysis of single-cell RNA-seq and bulk RNA-seq unravels T cell-related prognostic risk model and tumor immune microenvironment modulation in triple-negative breast cancer. Comput. Biol. Med..

[15] Wang K., Xiao Y., Zheng R., Cheng Y. (2024). Immune cell infiltration and drug response in glioblastoma multiforme: Insights from oxidative stress-related genes. Cancer Cell Int..

[16] Liu J., Lee J., Salazar Hernandez M.A., Mazitschek R., Ozcan U. (2015). Treatment of obesity with celastrol. Cell.

[17] Tong M., Xu Z., Wang L., Chen H., Wan X., Xu H., Yang S., Tu Q. (2025). An analysis of prognostic risk and immunotherapy response of glioblastoma patients based on single-cell landscape and nitrogen metabolism. Neurobiol. Dis..

[18] Alnahhas I., Khan M.M., Shi W. (2025). What single-cell RNA sequencing taught us about MGMT expression in glioblastoma. Neuro-Oncol. Adv..

[19] Yang Y., Zhang G. (2023). Lysosome-Related Diagnostic Biomarkers for Pediatric Sepsis Integrated by Machine Learning. J. Inflamm. Res..

[20] Zhuang C., Liu Y., Gu R., Du S., Long Y. (2023). Prognostic signature of colorectal cancer based on uric acid-related genes. Heliyon.

[21] Love M.I., Huber W., Anders S. (2014). Moderated estimation of fold change and dispersion for RNA-seq data with DESeq2. Genome Biol..

[22] Gustavsson E.K., Zhang D., Reynolds R.H., Garcia-Ruiz S., Ryten M. (2022). ggtranscript: An R package for the visualization and interpretation of transcript isoforms using ggplot2. Bioinformatics.

[23] Gu Z., Eils R., Schlesner M. (2016). Complex heatmaps reveal patterns and correlations in multidimensional genomic data. Bioinformatics.

[24] Zheng Y., Gao W., Zhang Q., Cheng X., Liu Y., Qi Z., Li T. (2022). Ferroptosis and Autophagy-Related Genes in the Pathogenesis of Ischemic Cardiomyopathy. Front. Cardiovasc. Med..

[25] Yu G., Wang L.G., Han Y., He Q.Y. (2012). clusterProfiler: An R package for comparing biological themes among gene clusters. Omics A J. Integr. Biol..

[26] Lei J., Qu T., Cha L., Tian L., Qiu F., Guo W., Cao J., Sun C., Zhou B. (2023). Clinicopathological characteristics of pheochromocytoma/paraganglioma and screening of prognostic markers. J. Surg. Oncol..

[27] Li Y., Lu F., Yin Y. (2022). Applying logistic LASSO regression for the diagnosis of atypical Crohn’s disease. Sci. Rep..

[28] Kamarudin A.N., Cox T., Kolamunnage-Dona R. (2017). Time-dependent ROC curve analysis in medical research: Current methods and applications. BMC Med. Res. Methodol..

[29] Zhang B., Xie L., Liu J., Liu A., He M. (2023). Construction and validation of a cuproptosis-related prognostic model for glioblastoma. Front. Immunol..

[30] Newman A.M., Liu C.L., Green M.R., Gentles A.J., Feng W., Xu Y., Hoang C.D., Diehn M., Alizadeh A.A. (2015). Robust enumeration of cell subsets from tissue expression profiles. Nat. Methods.

[31] Cantero D., Mollejo M., Sepúlveda J.M., D’Haene N., Gutiérrez-Guamán M.J., Rodríguez de Lope Á., Fiaño C., Castresana J.S., Lebrun L., Rey J.A. (2020). TP53, ATRX alterations, and low tumor mutation load feature IDH-wildtype giant cell glioblastoma despite exceptional ultra-mutated tumors. Neuro-Oncol. Adv..

[32] Mayakonda A., Lin D.C., Assenov Y., Plass C., Koeffler H.P. (2018). Maftools: Efficient and comprehensive analysis of somatic variants in cancer. Genome Res..

[33] Sachs M.C. (2017). plotROC: A Tool for Plotting ROC Curves. J. Stat. Softw..

[34] Heagerty P.J., Lumley T., Pepe M.S. (2000). Time-dependent ROC curves for censored survival data and a diagnostic marker. Biometrics.

[35] Hao Y., Hao S., Andersen-Nissen E., Mauck W.M., Zheng S., Butler A., Lee M.J., Wilk A.J., Darby C., Zager M. (2021). Integrated analysis of multimodal single-cell data. Cell.

[36] Liu X.H., Wang G.R., Zhong N.N., Zhu Z.R., Xiao Y., Li Z., Bu L.L., Liu B. (2025). Metal-dependent cell death resistance contribute to lymph node metastasis of oral squamous cell carcinoma. Front. Cell Dev. Biol..

[37] Jin S., Guerrero-Juarez C.F., Zhang L., Chang I., Ramos R., Kuan C.H., Myung P., Plikus M.V., Nie Q. (2021). Inference and analysis of cell-cell communication using CellChat. Nat. Commun..

[38] Wei W., Liu Y., Shen Y., Yang T., Dong Y., Han Z., Wang Y., Liu Z., Chai Y., Zhang M. (2024). In situ tissue profile of rat trigeminal nerve in trigeminal neuralgia using spatial transcriptome sequencing. Int. J. Surg..

[39] Chatsirisupachai K., Lagger C., de Magalhães J.P. (2022). Age-associated differences in the cancer molecular landscape. Trends Cancer.

[40] Murnan K.M., Horbinski C., Stegh A.H. (2023). Redox Homeostasis and Beyond: The Role of Wild-Type Isocitrate Dehydrogenases for the Pathogenesis of Glioblastoma. Antioxid. Redox Signal..

[41] Bailleul J., Vlashi E. (2023). Glioblastomas: Hijacking Metabolism to Build a Flexible Shield for Therapy Resistance. Antioxid. Redox Signal..

[42] Olivier C., Oliver L., Lalier L., Vallette F.M. (2020). Drug Resistance in Glioblastoma: The Two Faces of Oxidative Stress. Front. Mol. Biosci..

[43] Khatami S.H., Karami N., Taheri-Anganeh M., Taghvimi S., Tondro G., Khorsand M., Soltani Fard E., Sedighimehr N., Kazemi M., Rahimi Jaberi K. (2023). Exosomes: Promising Delivery Tools for Overcoming Blood-Brain Barrier and Glioblastoma Therapy. Mol. Neurobiol..

[44] Yabo Y.A., Niclou S.P., Golebiewska A. (2022). Cancer cell heterogeneity and plasticity: A paradigm shift in glioblastoma. Neuro-Oncology.

[45] Bacher M., Schrader J., Thompson N., Kuschela K., Gemsa D., Waeber G., Schlegel J. (2003). Up-regulation of macrophage migration inhibitory factor gene and protein expression in glial tumor cells during hypoxic and hypoglycemic stress indicates a critical role for angiogenesis in glioblastoma multiforme. Am. J. Pathol..

[46] Zhao J., Wang X., Li B. (2025). The Bidirectional Mechanism of Uric Acid Levels on Alzheimer’s Disease: A Narrative Review. Int. J. Gen. Med..

[47] Hooper D.C., Scott G.S., Zborek A., Mikheeva T., Kean R.B., Koprowski H., Spitsin S.V. (2000). Uric acid, a peroxynitrite scavenger, inhibits CNS inflammation, blood-CNS barrier permeability changes, and tissue damage in a mouse model of multiple sclerosis. FASEB J. Off. Publ. Fed. Am. Soc. Exp. Biol..

[48] Chen H., Shi D., Guo C., Zhang W., Guo Y., Yang F., Wang R., Zhang J., Fang Z., Yan Y. (2024). Can uric acid affect the immune microenvironment in bladder cancer? A single-center multi-omics study. Mol. Carcinog..

[49] Friedmann-Morvinski D., Narasimamurthy R., Xia Y., Myskiw C., Soda Y., Verma I.M. (2016). Targeting NF-κB in glioblastoma: A therapeutic approach. Sci. Adv..

[50] Aaberg-Jessen C., Fogh L., Sørensen M.D., Halle B., Brünner N., Kristensen B.W. (2019). Overexpression of TIMP-1 and Sensitivity to Topoisomerase Inhibitors in Glioblastoma Cell Lines. Pathol. Oncol. Res. POR.

[51] Zhang F., Ye J., Zhu J., Qian W., Wang H., Luo C. (2024). Key Cell-in-Cell Related Genes are Identified by Bioinformatics and Experiments in Glioblastoma. Cancer Manag. Res..

[52] He Y., Døssing K.B.V., Rossing M., Bagger F.O., Kjaer A. (2024). uPAR (PLAUR) Marks Two Intra-Tumoral Subtypes of Glioblastoma: Insights from Single-Cell RNA Sequencing. Int. J. Mol. Sci..

[53] Fu Z., Chen Z., Ye J., Ji J., Ni W., Lin W., Lin H., Lu L., Zhu G., Xie Q. (2024). Identifying PLAUR as a Pivotal Gene of Tumor Microenvironment and Regulating Mesenchymal Phenotype of Glioblastoma. Cancers.

[54] Zeng F., Li G., Liu X., Zhang K., Huang H., Jiang T., Zhang Y. (2021). Plasminogen Activator Urokinase Receptor Implies Immunosuppressive Features and Acts as an Unfavorable Prognostic Biomarker in Glioma. Oncologist.

[55] Tong M., Tu Q., Wang L., Chen H., Wan X., Xu Z. (2025). Joint analysis of single-cell RNA sequencing and bulk transcriptome reveals the heterogeneity of the urea cycle of astrocytes in glioblastoma. Neurobiol. Dis..

[56] Norton E.S., Whaley L.A., Jones V.K., Brooks M.M., Russo M.N., Morderer D., Jessen E., Schiapparelli P., Ramos-Fresnedo A., Zarco N. (2024). Cell-specific cross-talk proteomics reveals cathepsin B signaling as a driver of glioblastoma malignancy near the subventricular zone. Sci. Adv..

[57] Memon A., Lee W.K. (2018). KLF10 as a Tumor Suppressor Gene and Its TGF-β Signaling. Cancers.

[58] Chang V.H., Chu P.Y., Peng S.L., Mao T.L., Shan Y.S., Hsu C.F., Lin C.Y., Tsai K.K., Yu W.C., Ch’ang H.J. (2012). Krüppel-like factor 10 expression as a prognostic indicator for pancreatic adenocarcinoma. Am. J. Pathol..

[59] Jia F., Zhang L., Jiang Z., Tan G., Wang Z. (2023). FZD1/KLF10-hsa-miR-4762-5p/miR-224-3p-circular RNAs axis as prognostic biomarkers and therapeutic targets for glioblastoma: A comprehensive report. BMC Med. Genom..

[60] Liu-Chittenden Y., Jain M., Gaskins K., Wang S., Merino M.J., Kotian S., Kumar Gara S., Davis S., Zhang L., Kebebew E. (2017). RARRES2 functions as a tumor suppressor by promoting β-catenin phosphorylation/degradation and inhibiting p38 phosphorylation in adrenocortical carcinoma. Oncogene.

[61] Pachynski R.K., Wang P., Salazar N., Zheng Y., Nease L., Rosalez J., Leong W.I., Virdi G., Rennier K., Shin W.J. (2019). Chemerin Suppresses Breast Cancer Growth by Recruiting Immune Effector Cells Into the Tumor Microenvironment. Front. Immunol..

[62] Wang H., Wang X., Xu L., Zhang J. (2023). RARRES2 is Downregulated in Isocitrate Dehydrogenase 1 Mutant Glioma Patients and Served as an Unfavorable Prognostic Factor of Glioma. World Neurosurg..

[63] Xu P., Yang J., Liu J., Yang X., Liao J., Yuan F., Xu Y., Liu B., Chen Q. (2018). Identification of glioblastoma gene prognosis modules based on weighted gene co-expression network analysis. BMC Med. Genom..

[64] Wang Y., Yang H., Li N., Wang L., Guo C., Ma W., Liu S., Peng C., Chen J., Song H. (2024). A Novel Ubiquitin Ligase Adaptor PTPRN Suppresses Seizure Susceptibility through Endocytosis of Na(V)1.2 Sodium Channels. Adv. Sci..

[65] Wang D., Tang F., Liu X., Fan Y., Zheng Y., Zhuang H., Chen B., Zhuo J., Wang B. (2021). Expression and Tumor-Promoting Effect of Tyrosine Phosphatase Receptor Type N (PTPRN) in Human Glioma. Front. Oncol..

[66] Zhang H., Bao M., Liao D., Zhang Z., Tian Z., Yang E., Luo P., Jiang X. (2023). Identification of INSRR as an immune-related gene in the tumor microenvironment of glioblastoma by integrated bioinformatics analysis. Med. Oncol..

[67] Huang R., Lu X., Sun X., Wu H. (2024). A novel immune cell signature for predicting glioblastoma after radiotherapy prognosis and guiding therapy. Int. J. Immunopathol. Pharmacol..

[68] Pekmez M., Mete Ş.B., Aksüt Y., Öğütcü İ., Baştürk F.N., Gerçek Y.C., Şengelen A. (2025). Fatty acid synthase inhibitor cerulenin attenuates glioblastoma progression by reducing EMT and stemness phenotypes, inducing oxidative and ER stress response, and targeting PI3K/AKT/NF-κB axis. Med. Oncol..

[69] He Y., Sun M.M., Zhang G.G., Yang J., Chen K.S., Xu W.W., Li B. (2021). Targeting PI3K/Akt signal transduction for cancer therapy. Signal Transduct. Target. Ther..

[70] Jiang H., Nace R., Ferguson C., Zhang L., Peng K.W., Russell S.J. (2025). Oncolytic cytomegaloviruses expressing EGFR-retargeted fusogenic glycoprotein complex and drug-controllable interleukin 12. Cell Rep. Med..

[71] Ye T., Cheng Y., Li C. (2021). Adaptor Protein Complex 1 Sigma 3 Is Highly Expressed in Glioma and Could Enhance Its Progression. Comput. Math. Methods Med..

[72] Jandial R., Neman J., Lim P.P., Tamae D., Kowolik C.M., Wuenschell G.E., Shuck S.C., Ciminera A.K., De Jesus L.R., Ouyang C. (2018). Inhibition of GLO1 in Glioblastoma Multiforme Increases DNA-AGEs, Stimulates RAGE Expression, and Inhibits Brain Tumor Growth in Orthotopic Mouse Models. Int. J. Mol. Sci..

[73] Yao P., Ju H., Song A., Wang Y., Xin G., Wang G., Ma J., Guo M. (2025). Ruxolitinib suppresses tumor growth in PTEN-deficient glioblastoma by inhibiting the STAT3-PDL1 axis-mediated the M2 polarization of macrophages. Int. Immunopharmacol..

[74] Rossi S., Giovannoni I., Patrizi S., Mafficcini A., Piccirilli E., Ricciardi G.K., Megaro G., Arienzo F., Tancredi C., Agolini E. (2025). Expanding clinicopathologic knowledge in high-grade glioma with pleomorphic and pseudopapillary features (HPAP): A report of two cases. Acta Neuropathol. Commun..

[75] Duplan E., Bernardin A., Goiran T., Leroudier N., Casimiro M., Pestell R., Tanaka S., Malleval C., Honnorat J., Idbaih A. (2025). α-synuclein expression in glioblastoma restores tumor suppressor function and rescues temozolomide drug resistance. Cell Death Dis..

[76] Su Z., Han S., Jin Q., Zhou N., Lu J., Shangguan F., Yu S., Liu Y., Wang L., Lu J. (2021). Ciclopirox and bortezomib synergistically inhibits glioblastoma multiforme growth via simultaneously enhancing JNK/p38 MAPK and NF-κB signaling. Cell Death Dis..

[77] Larsson P., Olsson M., Sarathchandra S., Fäldt Beding A., Forssell-Aronsson E., Kovács A., Karlsson P., Helou K., Parris T.Z. (2024). Multi-omics analysis identifies repurposing bortezomib in the treatment of kidney-, nervous system-, and hematological cancers. Sci. Rep..

[78] Kulagin K.A., Starodubova E.S., Osipova P.J., Lipatova A.V., Cherdantsev I.A., Poddubko S.V., Karpov V.L., Karpov D.S. (2024). Synergistic Effect of a Combination of Proteasome and Ribonucleotide Reductase Inhibitors in a Biochemical Model of the Yeast Saccharomyces cerevisiae and a Glioblastoma Cell Line. Int. J. Mol. Sci..

[79] Hu S., Chen G., Luo A., Zhao H., Li D., Peng B., Du J., Luo D. (2025). Mechanism of LINC01018/miR-182-5p/Rab27B in the immune escape through PD-L1-mediated CD8^+^ T cell suppression in glioma. Biol. Direct.

[80] Faust Akl C., Andersen B.M., Li Z., Giovannoni F., Diebold M., Sanmarco L.M., Kilian M., Fehrenbacher L., Pernin F., Rone J.M. (2025). Glioblastoma-instructed astrocytes suppress tumour-specific T cell immunity. Nature.

[81] Alrefai H., Lin B., Elkohly A., Kumar M., Schanel T.L., Lee K.J., Hicks P.H., Anderson J.C., Guo G., Ahn E.E. (2025). Xenoline-polarized macrophages as a physiologically relevant in vitro model of tumor-associated macrophages in glioblastoma. Res. Sq..

[82] Yan T., Yang H., Meng Y., Li H., Jiang Q., Liu J., Xu C., Xue Y., Xu J., Song Y. (2023). Targeting copper death genotyping associated gene RARRES2 suppresses glioblastoma progression and macrophages infiltration. Cancer Cell Int..

[83] Wu J., Shen S., Liu T., Ren X., Zhu C., Liang Q., Cui X., Chen L., Cheng P., Cheng W. (2022). Chemerin enhances mesenchymal features of glioblastoma by establishing autocrine and paracrine networks in a CMKLR1-dependent manner. Oncogene.

[84] Li P., Chen F., Yao C., Zhu K., Zhang B., Zheng Z. (2022). PTPRN Serves as a Prognostic Biomarker and Correlated with Immune Infiltrates in Low Grade Glioma. Brain Sci..

