# Supplementary figures and images for: Prognostic Role of Uric Acid-Related Gene Signatures in Glioblastoma Multiforme: Insights from Bulk RNA and Single-Cell RNA Sequencing
Source: Cancers (Basel). 2026 Apr 20;18(8):1297. doi: 10.3390/cancers18081297 (PMC13114913; doi:10.3390/cancers18081297)

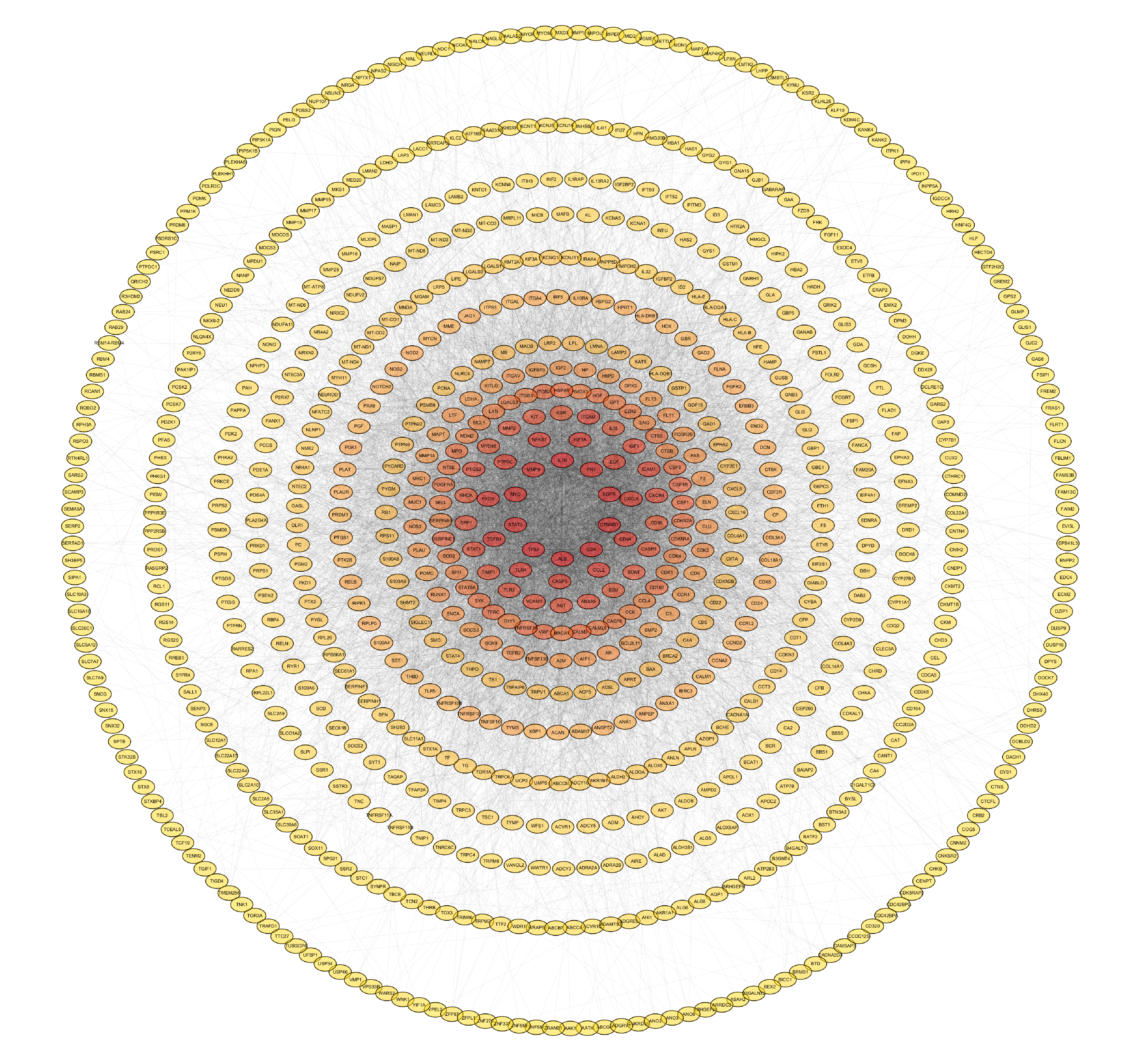

Supplement: Supplementary file 1 [file cancers-18-01297-s001.zip › Figure S1.tif]

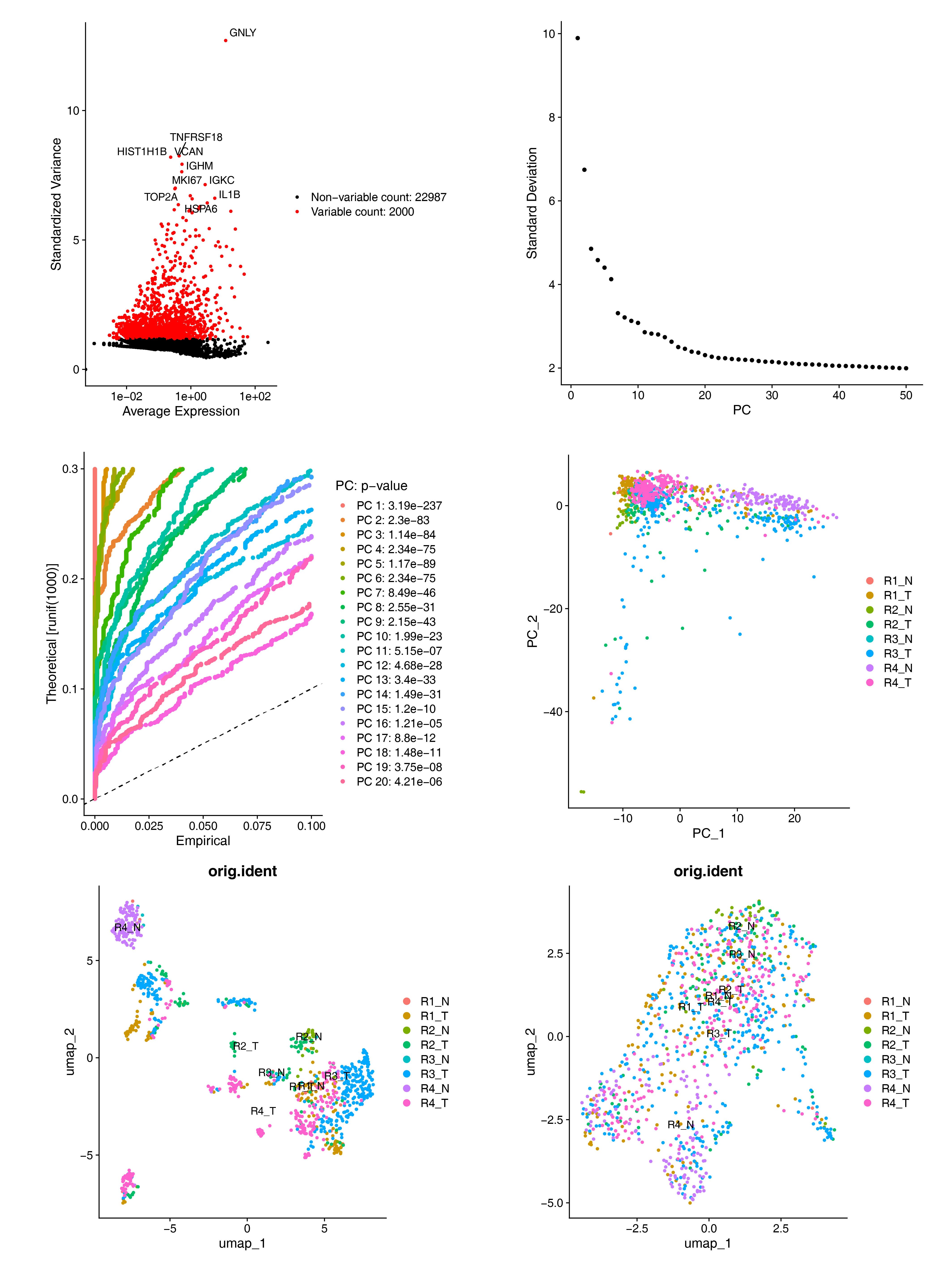

Supplement: Supplementary file 1 [file cancers-18-01297-s001.zip › Figure S10.tif]

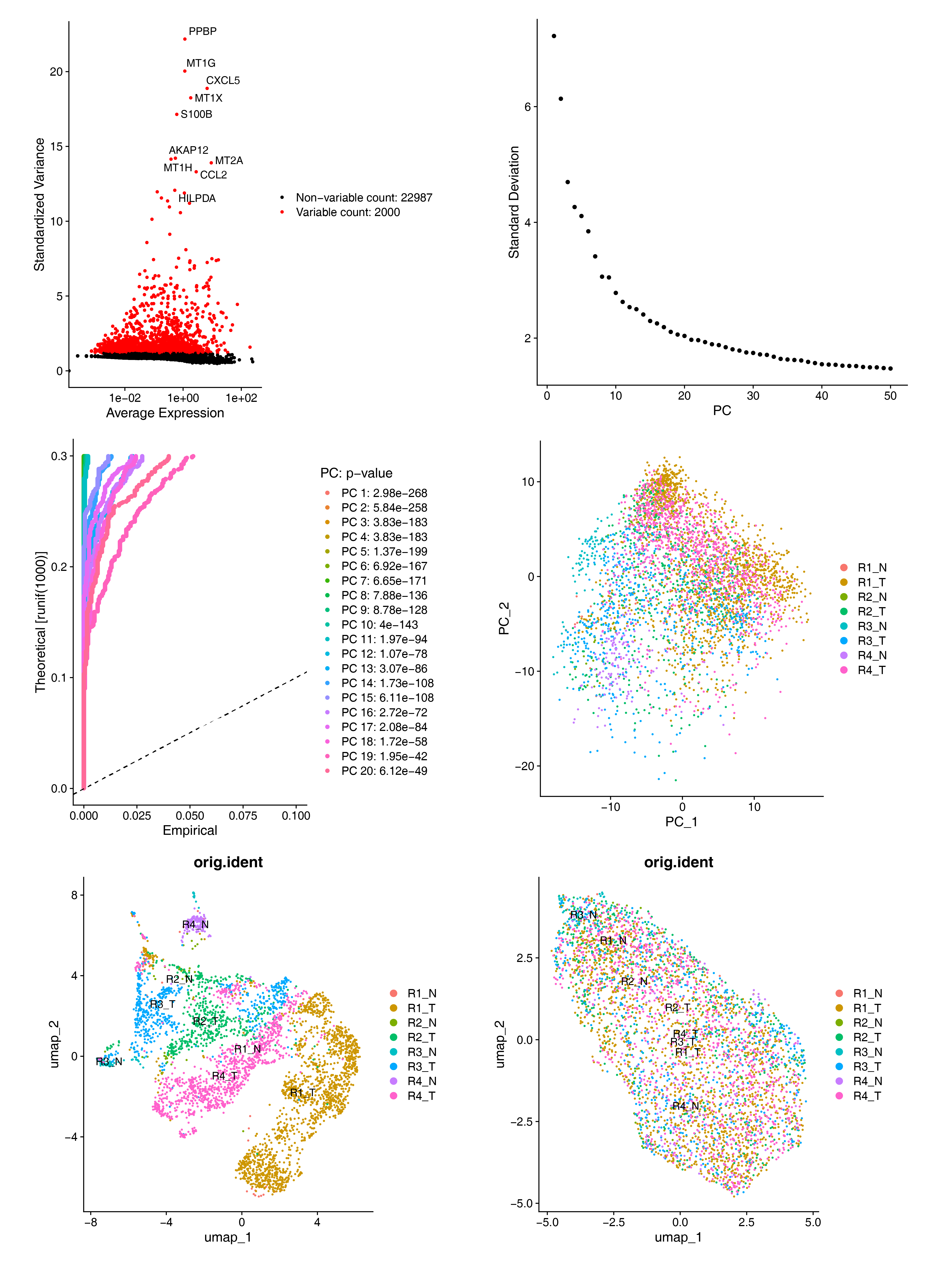

Supplement: Supplementary file 1 [file cancers-18-01297-s001.zip › Figure S11.tif]

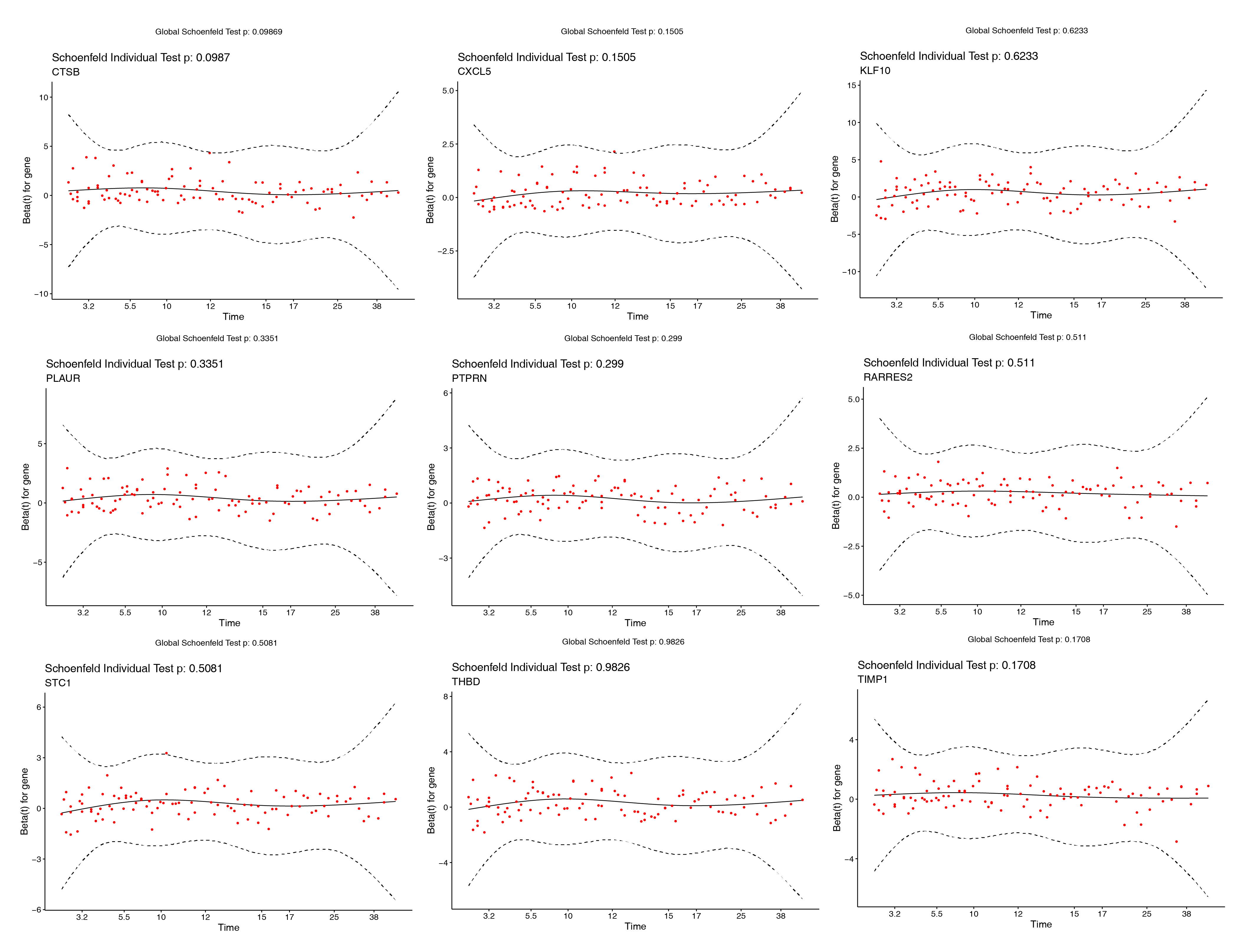

Supplement: Supplementary file 1 [file cancers-18-01297-s001.zip › Figure S2.tif]

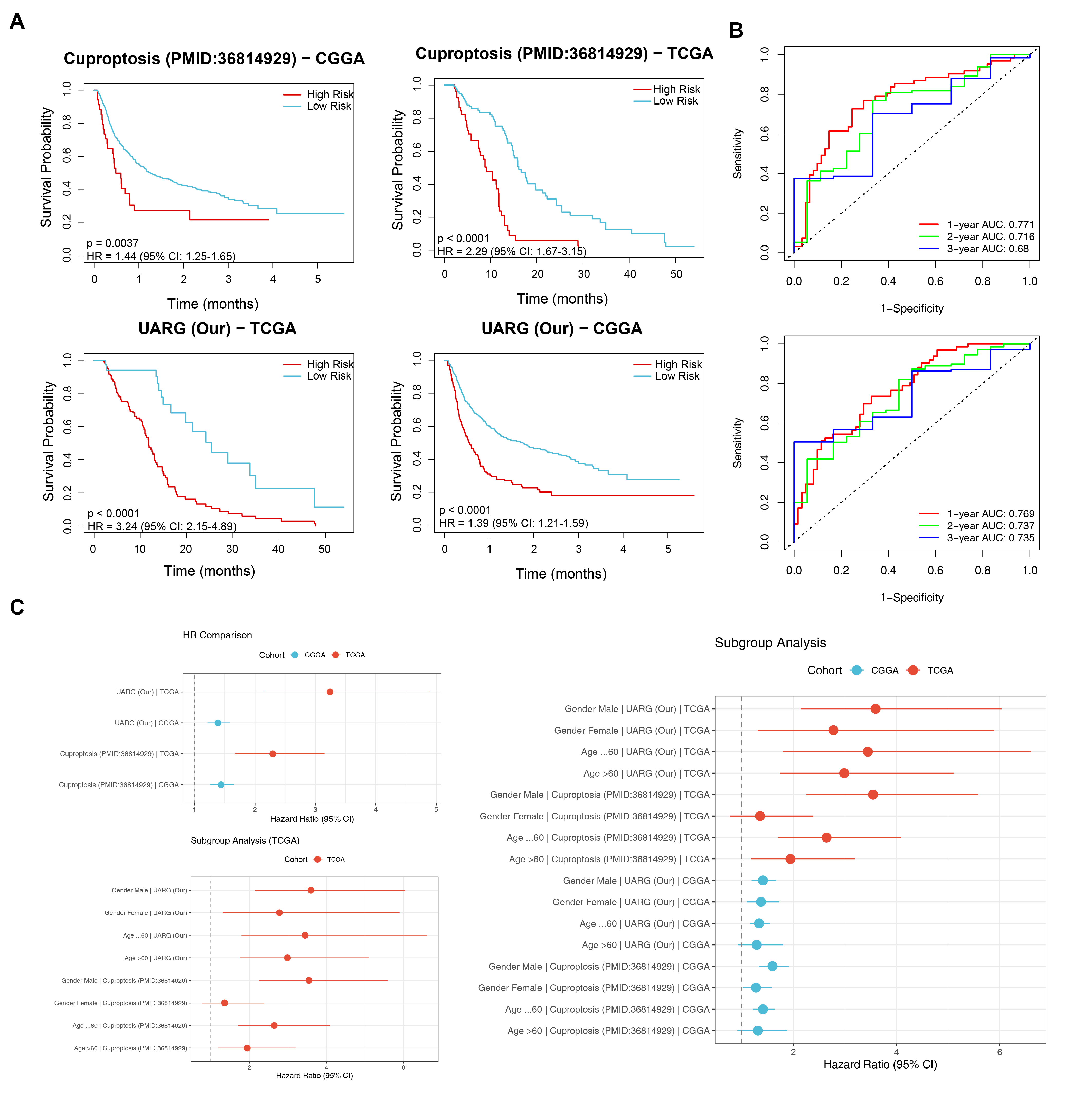

Supplement: Supplementary file 1 [file cancers-18-01297-s001.zip › Figure S3.tif]

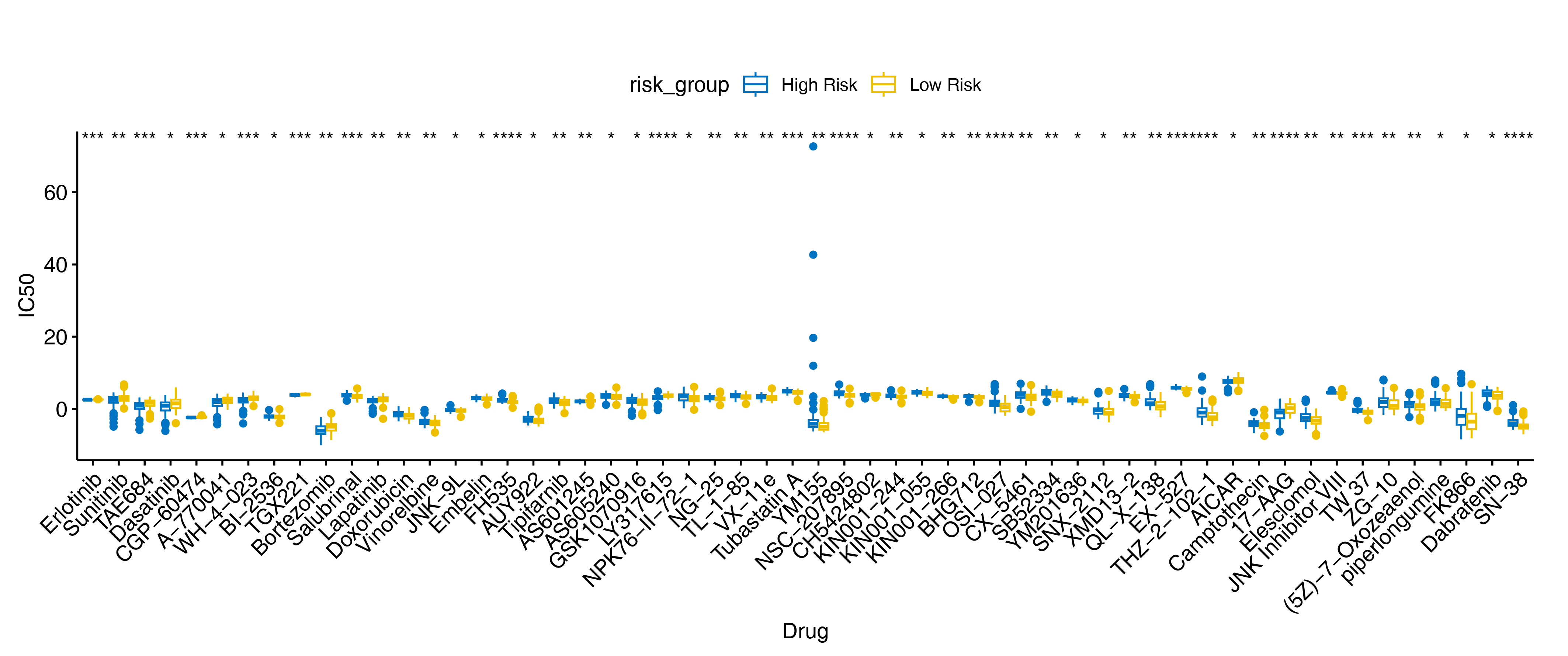

Supplement: Supplementary file 1 [file cancers-18-01297-s001.zip › Figure S4.tif]

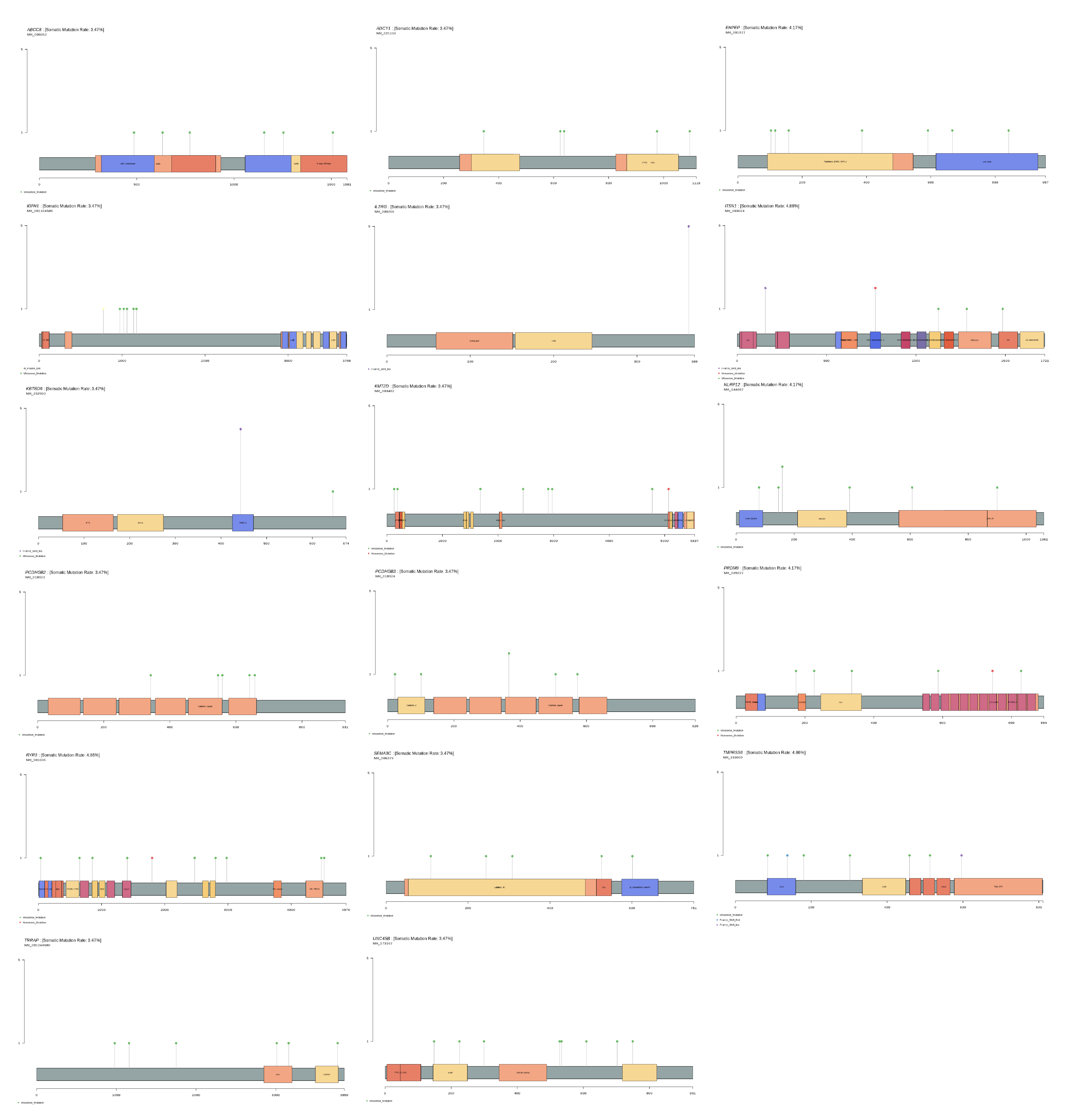

Supplement: Supplementary file 1 [file cancers-18-01297-s001.zip › Figure S5.tif]

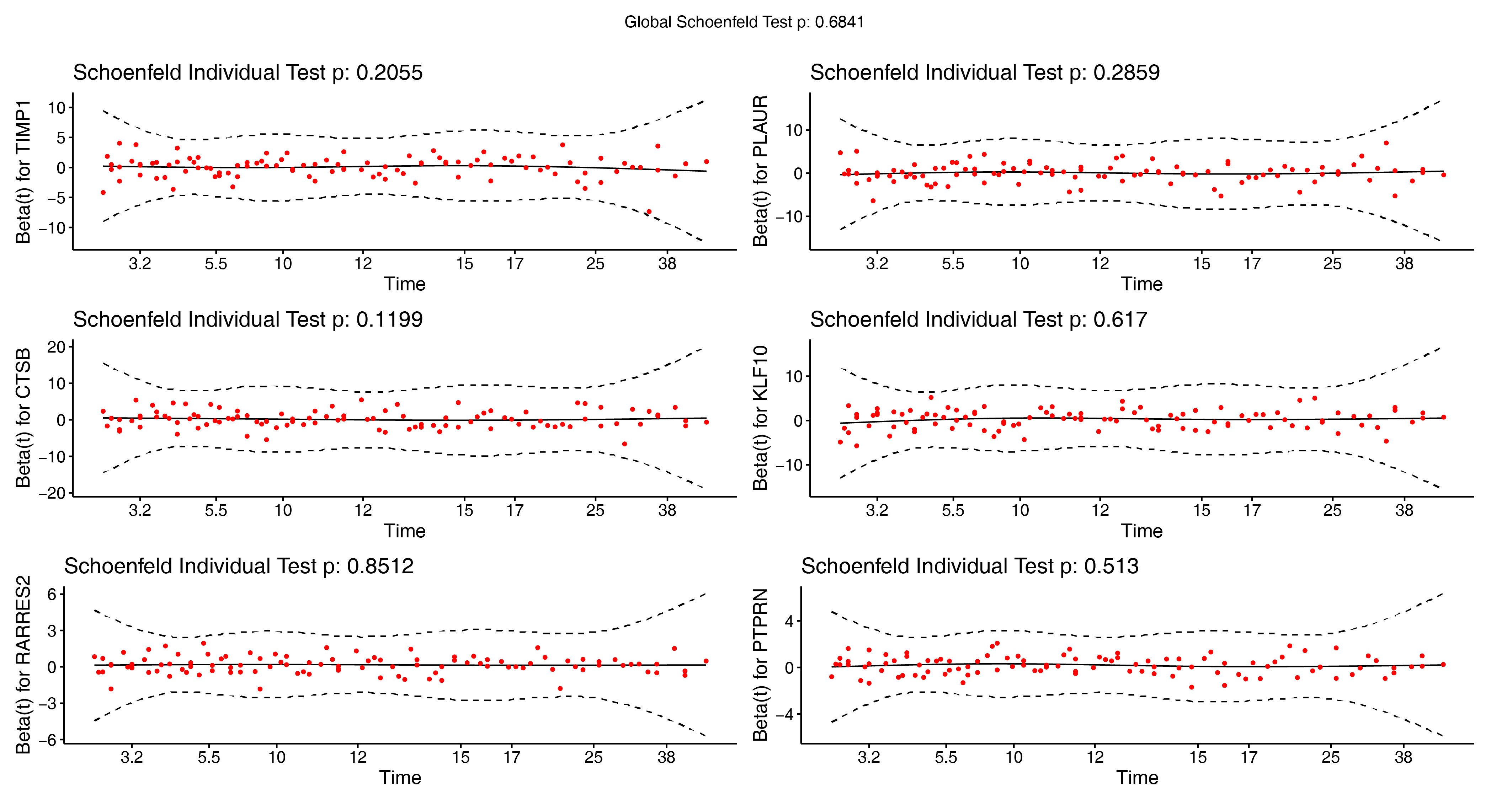

Supplement: Supplementary file 1 [file cancers-18-01297-s001.zip › Figure S6.tif]

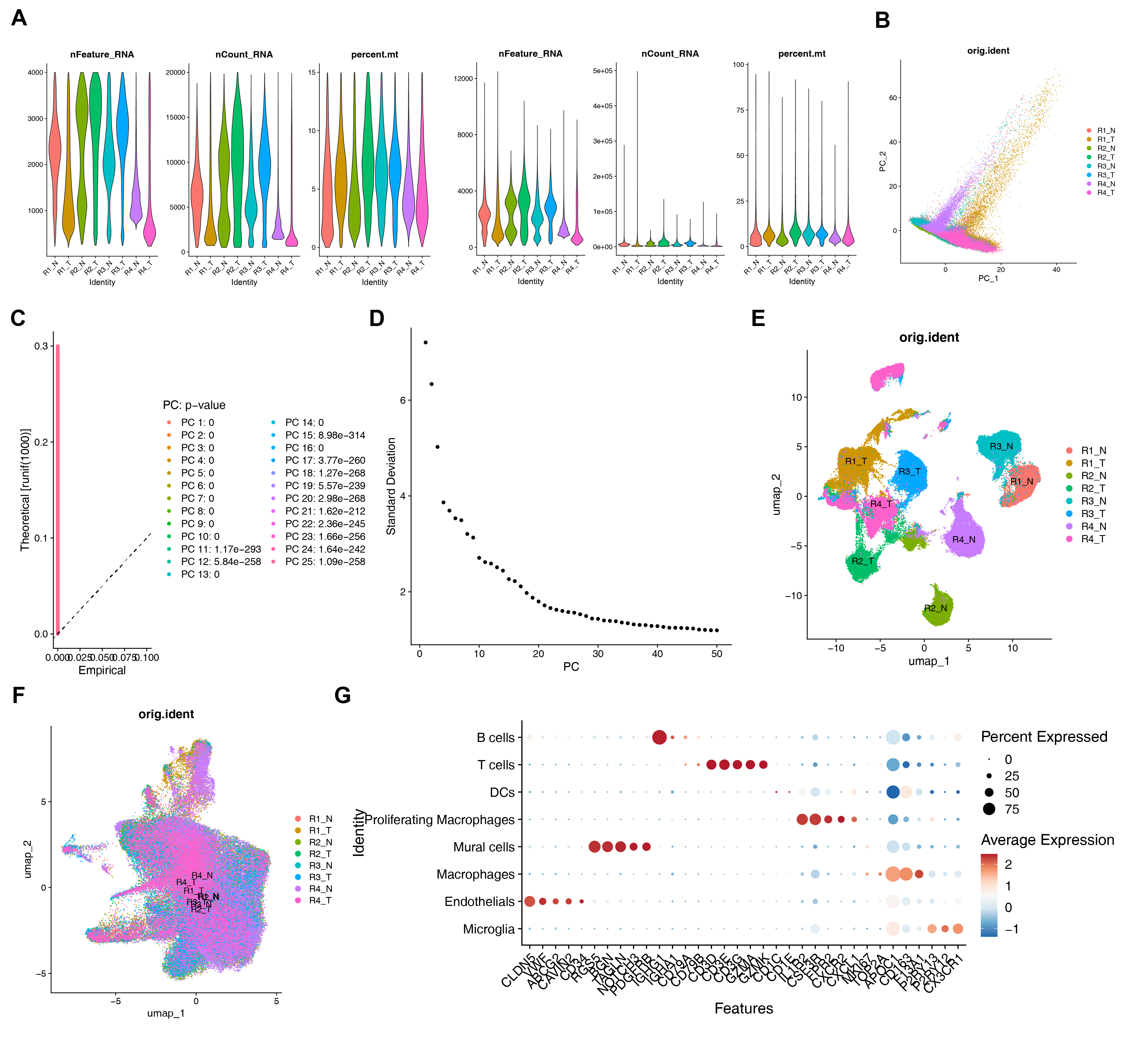

Supplement: Supplementary file 1 [file cancers-18-01297-s001.zip › Figure S7.tif]

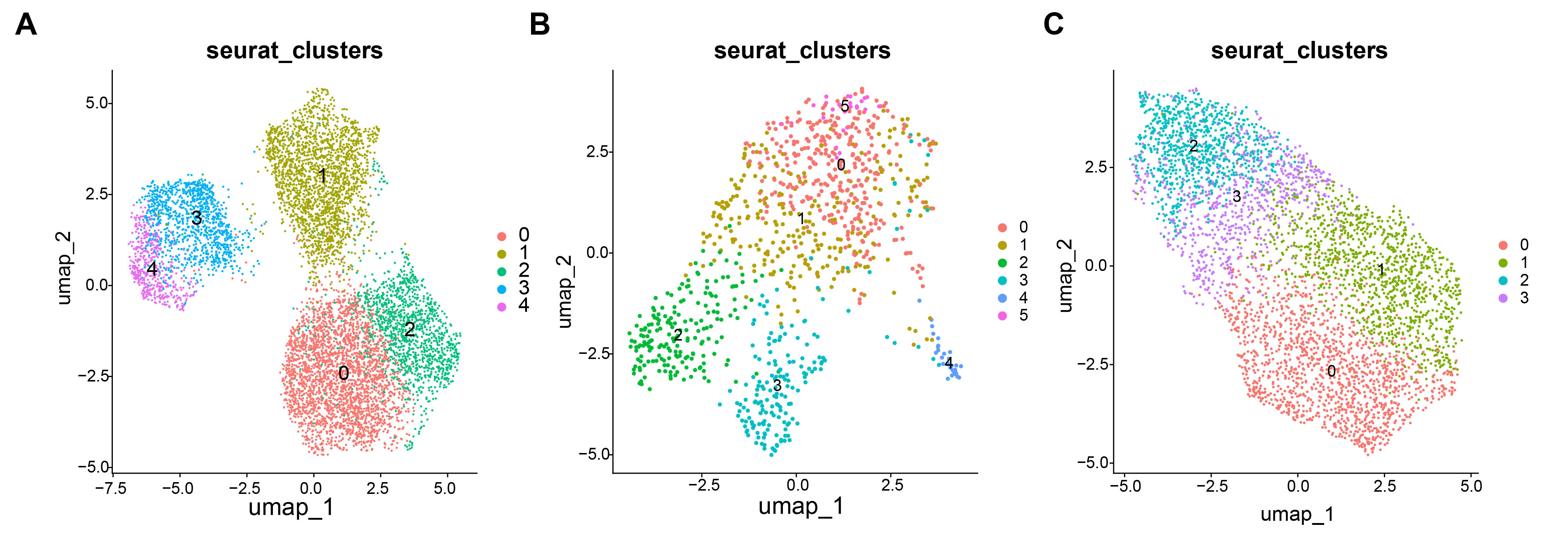

Supplement: Supplementary file 1 [file cancers-18-01297-s001.zip › Figure S8.tif]

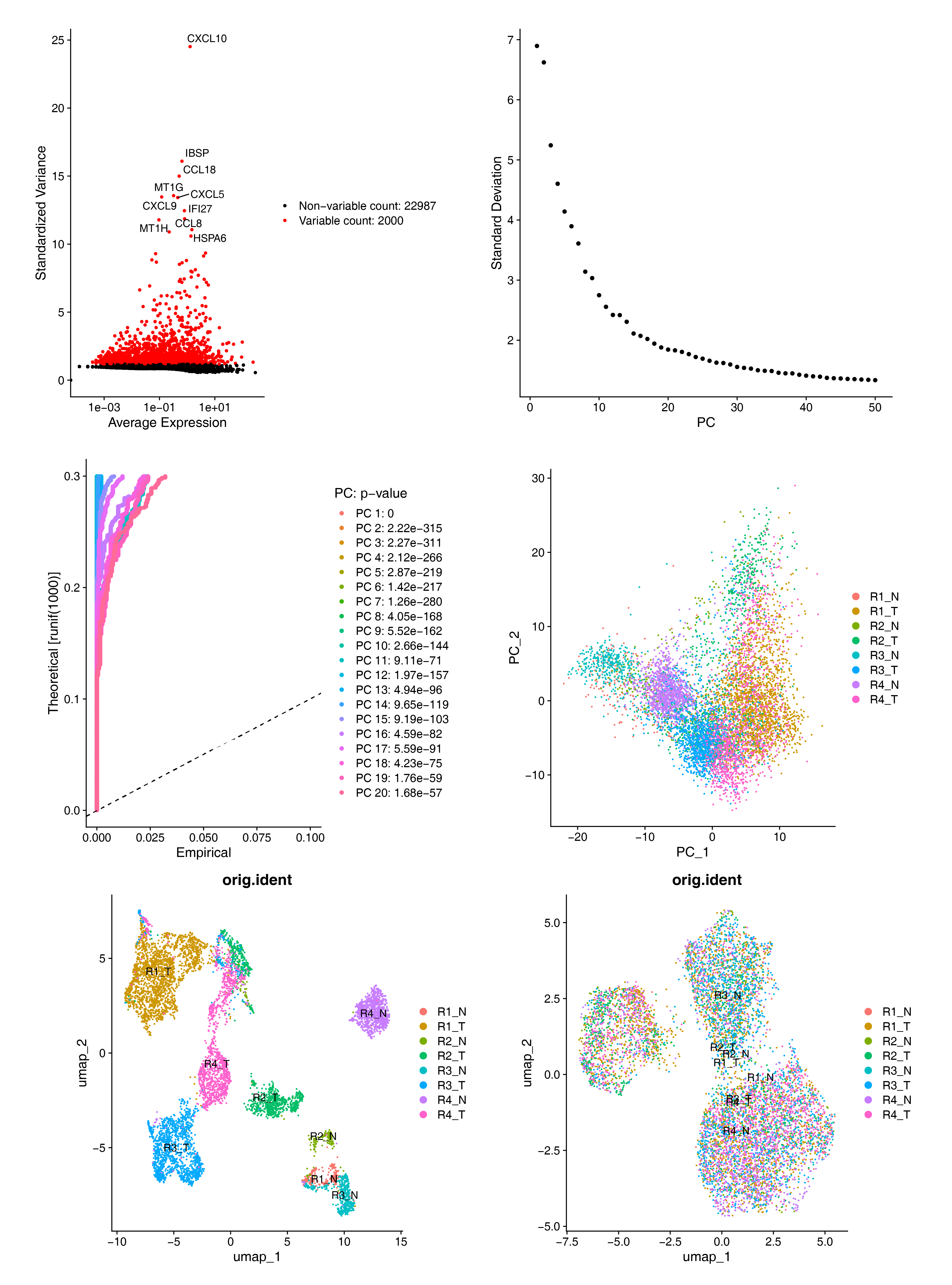

Supplement: Supplementary file 1 [file cancers-18-01297-s001.zip › Figure S9.tif]
